# Trends in disease burden and risk factors of asthma from 1990 to 2019 in Belt and Road Initiative countries: evidence from the Global Burden of Disease Study 2019

**DOI:** 10.1080/07853890.2024.2399964

**Published:** 2024-09-06

**Authors:** Wenjing Ye, Xue Xu, Yibo Ding, Xiaopan Li, Wen Gu

**Affiliations:** a Department of Respiratory Medicine, School of Medicine, Xinhua Hospital, Shanghai Jiao Tong University, Shanghai, China; b Department of Epidemiology, Naval Medical University, Shanghai, China; c Department of Health Management Center, Zhongshan Hospital, Shanghai Medical College of Fudan University, Shanghai, China

**Keywords:** ‘B&R’ countries, asthma burden, burden of disease, years lived with disability, risk factor

## Abstract

This study outlines asthma burden trends across age, sex, regions and risk factors in ‘Belt and Road’ (B&R) countries from 1990 to 2019 using the Global Burden of Disease Study 2019 data. Incidence, mortality, prevalence, years lived with disability (YLDs), disability-adjusted life years (DALYs) and risk factors for asthma were measured. India, China and Indonesia bore the heaviest burden in 2019. Despite the significant decline in the average annual percent change for age-standardized mortality and years of life lost from 1990 to 2019, increases were observed in several East Asian, Central Asian, North African and Middle Eastern countries between 2010 and 2019. For both sexes, YLDs decreased in most B&R countries but increased in Montenegro, Saudi Arabia, Armenia, Vietnam and Oman. YLDs in Georgia, the United Arab Emirates and Albania increased in males but decreased in females. YLDs increased for those aged <15 years in Central Asia and Europe, while China’s 50–74-year age group showed the lowest YLD change. High body mass index (BMI) led to increased YLDs in East, Central and Southeast Asia; North Africa; and the Middle East. Conclusively, asthma burden varies significantly by country. Tailoring control efforts to specific regions, sex and high BMI could enhance asthma management.

## Introduction

The ‘Belt and Road’ (B&R) initiative, proposed by China in 2013, refers to the Silk Road Economic Belt and the twenty-first century Maritime Silk Road. Health is a fundamental prerequisite for economic and social development. The strategy of the B&R initiative targets the growth of economic trade and investment and aspires to foster integration in the health sector. The B&R initiative covers countries with a total population exceeding 4.4 billion, stretching from the Asia-Pacific to Europe, the Middle East, Africa and the South Pacific [1]. These diverse regions and countries have varying degrees of economic development, healthcare threats and disease burdens. To make the B&R a viable project, member countries of the B&R initiative must strengthen close cooperation within the health system.

Asthma, a common chronic respiratory disease characterized by chronic airway inflammation, is a serious public health challenge that affects 1–29% of the population across all age groups [2,3]. Asthma poses a substantial human and economic burden worldwide. In 2019, the European Union members allocated approximately €380 billion to care for patients with chronic respiratory diseases [4]. Factors such as population aging, sex, economic resources, healthcare, medical technology and housing conditions are associated with asthma’s prevalence and mortality rate [5,6]. Most asthma-related deaths occur in lower- and middle-income countries (LMICs), mainly because of under-diagnosis and under-treatment.

The Global Burden of Disease Study (GBD) 2019 comprehensively evaluated the incidence, mortality and age- and sex-related differences in all causes and specific causes of 369 diseases and injuries across 204 countries and regions between 1990 and 2019. Incidence, mortality, years lived with disability (YLDs) and disability-adjusted life years (DALYs) have become comprehensive indicators of health loss.

China’s B&R initiative, health development assistance and investment funds are harmoniously coming together to form a unique global impact, substantially influencing global health. This initiative encompasses various activities such as providing training for healthcare workers, establishing disease control facilities, and developing networks for medical research and knowledge exchange [7]. In the context of the COVID-19 pandemic, the B&R Initiative serves as a significant platform enabling member countries to share and discuss clinical treatment protocols and strategies for disease containment [8]. However, only a few studies have focused on the burden of asthma in B&R member countries, and this study stands out as one of them. We assume that examining the varying trends in the disease burden of asthma and the risk factors associated with asthma, can help gauge the overall status of asthma and seek to develop a suitable model for mutual health aid, which adds to the novelty of this study.

## Materials and methods

### Data source

The methods employed in the GBD 2019 have been extensively reported elsewhere [9]. As per GBD 2019, asthma was defined based on self-reported wheezing and a physician’s diagnosis within the past 12 months. This definition corresponds with Code 493 in the 9th revision of the International Classification of Diseases and Codes J45 and J46 in its 10th revision [5].

### B&R countries

This study encompasses 66 countries participating in the B&R initiative, spread across several regions: East Asia, South Asia, Southeast Asia, Central Asia, the Middle East, North Africa, Central Europe, Eastern Europe and Western Europe. The geographical distribution of these member countries is depicted in Figure S1. The socio-demographic index (SDI) is a comprehensive measure of a region’s economic development, with a higher index denoting a more advanced economy [9]. The regions were divided into five categories: low (0–0.455), low-middle (0.456–0.608), middle (0.609–0.690), high-middle (0.691–0.805) and high (0.806–1). The methods of SDI development and computation are detailed elsewhere [5,10].

### Estimation of asthma burden

Age-standardized rates of incidence, mortality and prevalence, as well as YLDs and DALYs in the 66 countries and regions that were part of the B&R initiative from 1990 to 2019 were assessed. YLDs were estimated by multiplying the prevalence of each sequela by the disability weight associated with the respective condition, including an adjustment for coexisting conditions or comorbidities.

The years of life lost (YLLs) were determined by multiplying the number of deaths by the remaining life expectancy according to the GBD standard life table. DALYs are the summation of YLLs and YLDs. To promote transparency and replicability in our study, we adhered to the Guidelines for Accurate and Transparent Health Estimates Reporting (GATHER) [11].

### Statistical analysis

Age-standardized estimates facilitate comparisons over time and across countries by adjusting for variations in population age distribution. The Joinpoint Regression Program (Version 4.9.0.0, March 2021) was utilized to evaluate trends in disease burden from 1990 to 2019, calculated as the average annual percentage change (AAPC) [12]. Uncertainty intervals (UIs) were defined as the 2.5th and 97.5th percentile values within the posterior distributions. Differences between estimates were calculated using a 1000-draw level. We generated 95% UIs for all reported data. All differences >95% of the values were considered significant [13]. A *p* value <.05 was considered statistically significant.

## Results

### Absolute numbers for incidence, mortality, prevalence, YLDs, YLLs and DALYs due to asthma in 2019

The absolute numbers for incidence, mortality, prevalence, YLDs, YLLs and DALYs attributed to asthma in 2019 are presented in Table S1, categorized by each B&R member country. There are notable variations in the disease burden of asthma across different countries. Among them, India, China and Indonesia emerged as the three nations with the highest asthma burden. Specifically in India, there were 1305163.77 (95% UI: 839030.54–1930247.47) YLDs, 4533600.98 (95% UI: 3082605.36–5949567.82) YLLs and 5838764.76 (95% UI: 4244934.47–7429627.78) DALYs due to asthma in 2019. On the other hand, the country with the lowest DALYs was the Maldives in Southeast Asia, reporting 660.23 (95% UI: 485.32–900.22) DALYs.

### Disease burden of asthma in 1990 and 2019

**Figure 1.**
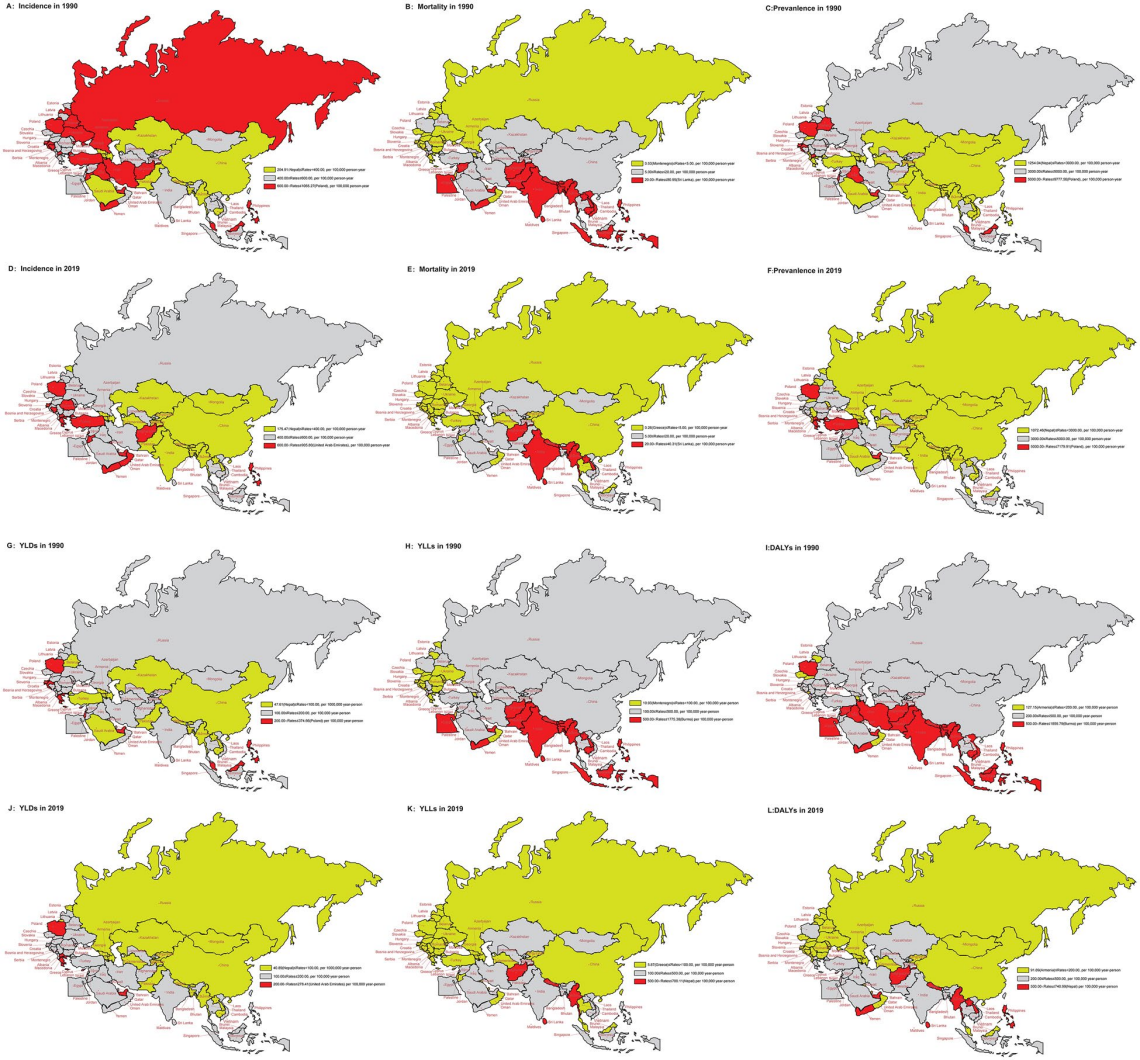
Age-standardized rates of incidence, mortality, prevalence, YLLs, YLDs and DALYs in 1990 and 2019 in “Belt & Road” countries. (A) Age-standardized incidence rate in 1990; (B) age-standardized mortality rate in 1990; (C) age-standardized prevalence rate in 1990; (D) age-standardized incidence rate in 2019; (E) age-standardized mortality rate in 2019; (F) age-standardized prevalence rate in 2019; (G) age-standardized YLDs in 1990; (H) age-standardized YLLs in 1990; (I) age-standardized DALYs in 1990; (J) age-standardized YLDs rate in 2019; (K) age-standardized YLLs in 2019; (L) age-standardized DALYs in 2019.

Low-SDI regions exhibited higher age-standardized mortality rates and lower age-standardized incidence rates. In contrast, high-SDI regions showed lower mortality rates and higher incidence rates of asthma in 1990 and 2019. The incidence, mortality, prevalence, YLDs, YLLs and DALYs for asthma were lower in 2019 than in 1990 (Figure 1, Table S2). In 1990, Poland had the highest incidence, prevalence and YLDs (1065.27 per 100,000, 9777.56 per 100,000 and 374.66 per 100,000, respectively), and Burma had the highest YLLs (141.71 per 100,000) and DALYs (516.38 per 100,000). However, in 2019, the United Arab Emirates recorded the highest incidence, prevalence and YLDs (905.80 per 100,000, 7179.91 per 100,000 and 278.41 per 100,000, respectively). Nepal, on the other hand, had the highest YLLs (700.11 per 100,000) and DALYs (740.99 per 100,000).

### Trends in age-standardized incidence, prevalence, mortality, YLLs and DALYs

**Figure 2.**
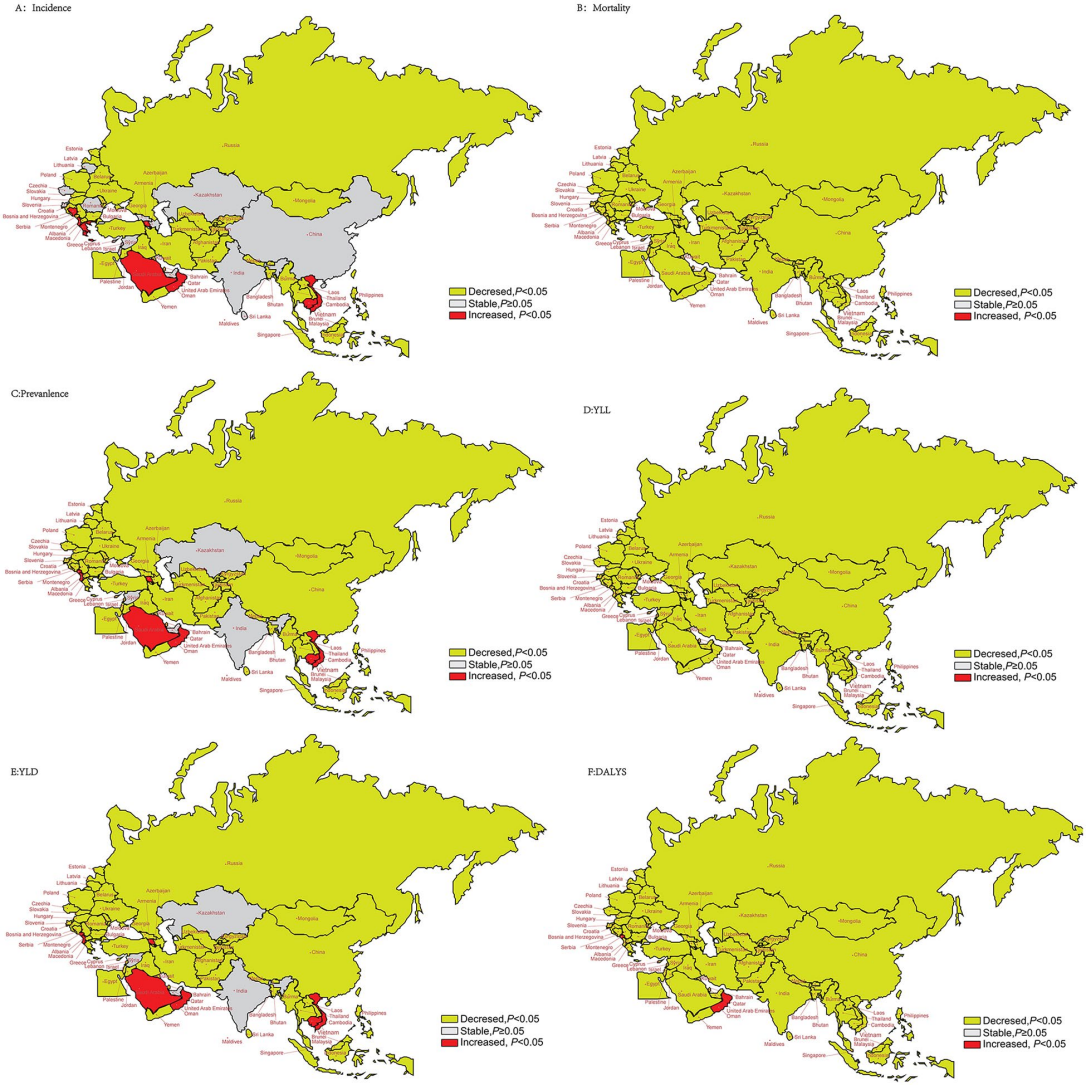
Trends of age-standardized rates of incidence, mortality and prevalence of YLLs, YLDs and DALYs in 1990–2019 in “Belt & Road” countries. (A) AAPC of age-standardized incidence rate; (B) AAPC of age-standardized mortality rate; (C) AAPC of age-standardized prevalence rate; (D) AAPC of age-standardized YLLs; (E) AAPC of age-standardized YLDs; (F) AAPC of age-standardized DALYs.

Figure 2(B,D) and Table S3 show a consistent downward trend in the AAPC of age-standardized mortality and YLLs across all B&R member countries from 1990 to 2019 (*p* < .001). Notably, a statistically significant increasing trend in the AAPC of age-standardized incidence and prevalence was observed in Vietnam, Saudi Arabia, Albania, Armenia, Cambodia and Oman. Conversely, most of other countries experienced either a decreasing trend or no significant change (Figure 2(A,C)). Regarding age-standardized DALYs, an increase was observed only in Oman and Montenegro by 0.36% (95% CI: 0.12–0.61%, *p* < .001) and 0.17% (95% CI: 0.05–0.28%, *p* < .001), respectively (Figure 2(F)).

### Trends in age-standardized YLDs

**Figure 3.**
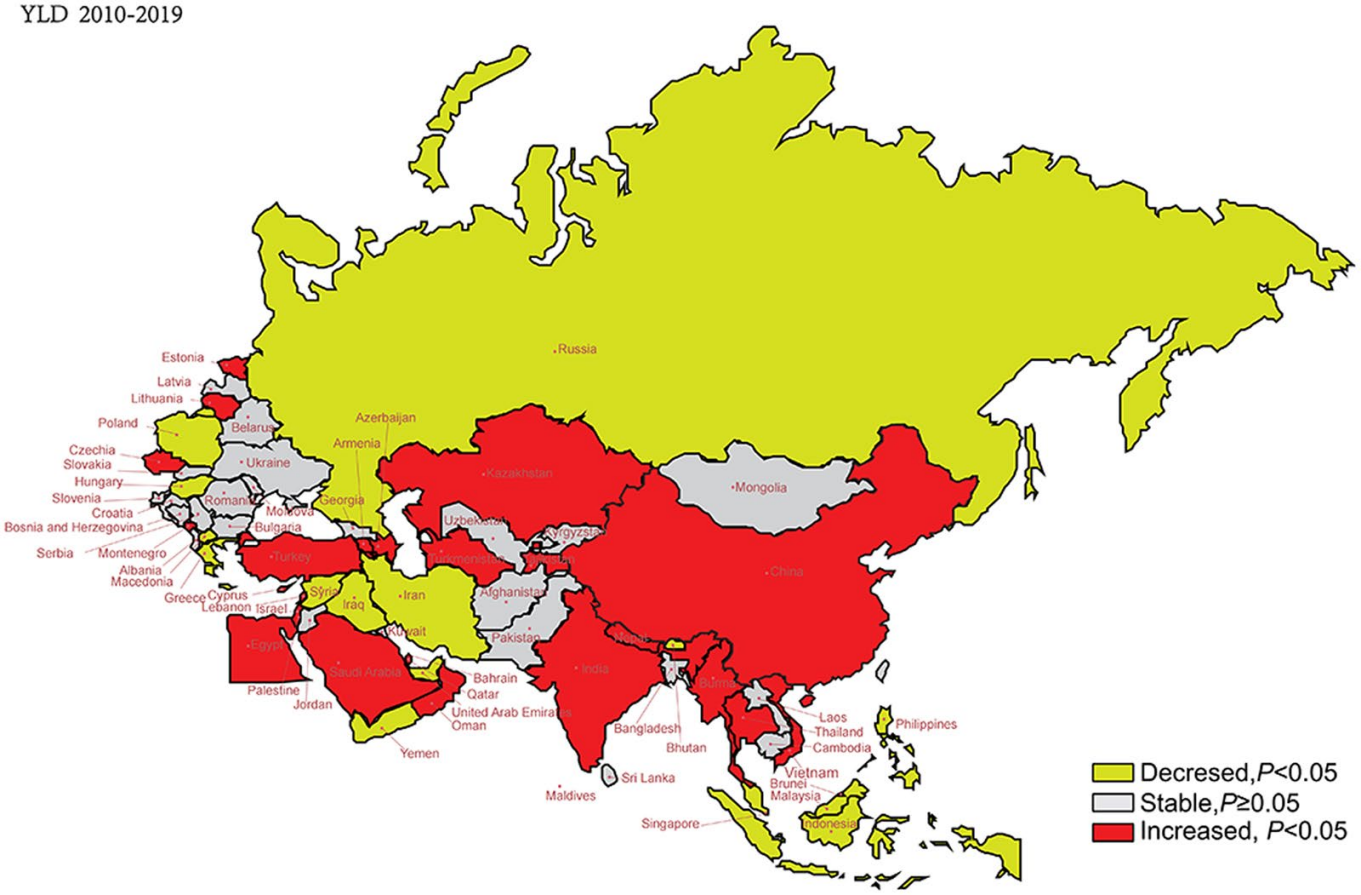
Trends of age-standardized rates of YLDs in 2010–2019 in “Belt & Road” countries.

Figures 2(E) and 3 show the changing trends in age-standardized YLDs in the B&R member countries for 1990–2019 and 2010–2019. Differences in the changing trends between these two periods were observed. Except for Montenegro, Saudi Arabia, Albania, Armenia, Cambodia, Vietnam and Oman, the other member countries exhibited a downward trend in the AAPC of age-standardized YLDs from 1990 to 2019. However, from 2010 to 2019, more countries experienced an upward trend in the AAPC of age-standardized YLDs, mainly in East Asia, Central Asia, North Africa and the Middle East. Particularly, China showed a significant increase in the AAPC of age-standardized YLDs by 4.74% (95% CI: 2.63–6.88%, *p* < .001). Table S4 provides further details.

### Trends in age-standardized YLDs stratified by gender and age groups

**Figure 4.**
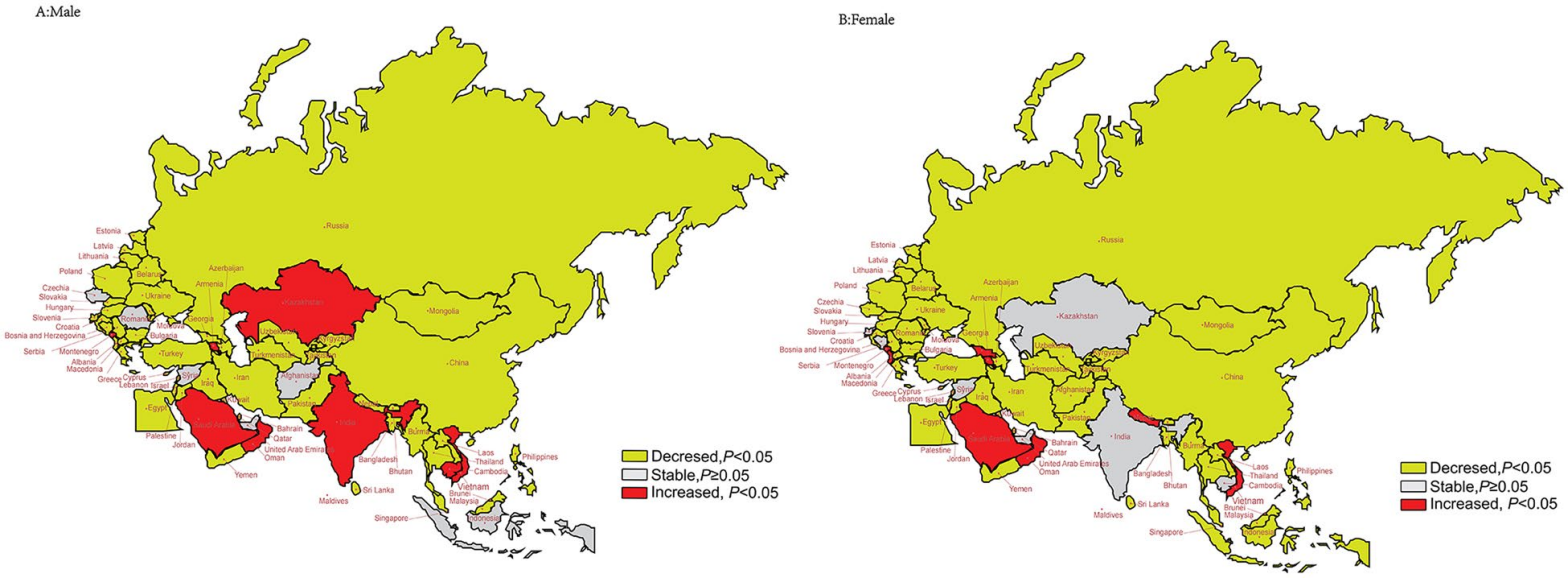
Trends of age-standardized rates of YLDs by gender in 1990–2019 in “Belt & Road” countries. (A) AAPC of age-standardized rates of YLDs in males from 1990 to 2019; (B) AAPC of age-standardized rates of YLDs in females from 1990 to 2019.

Sex differences in AAPC trends in age-standardized YLD from 1990 to 2019 were observed (Figure 4, Table S5). For both sexes, most B&R member countries exhibited a consistent decrease in trend, whereas Montenegro, Saudi Arabia, Armenia, Vietnam and Oman showed an increase in trend (*p* < .05). Conversely, Georgia, the United Arab Emirates and Albania showed an upward trend in males and a downward trend in females (*p* < .05). No significant change in trend was observed for males and females in the Syrian Arab Republic.

**Figure 5.**
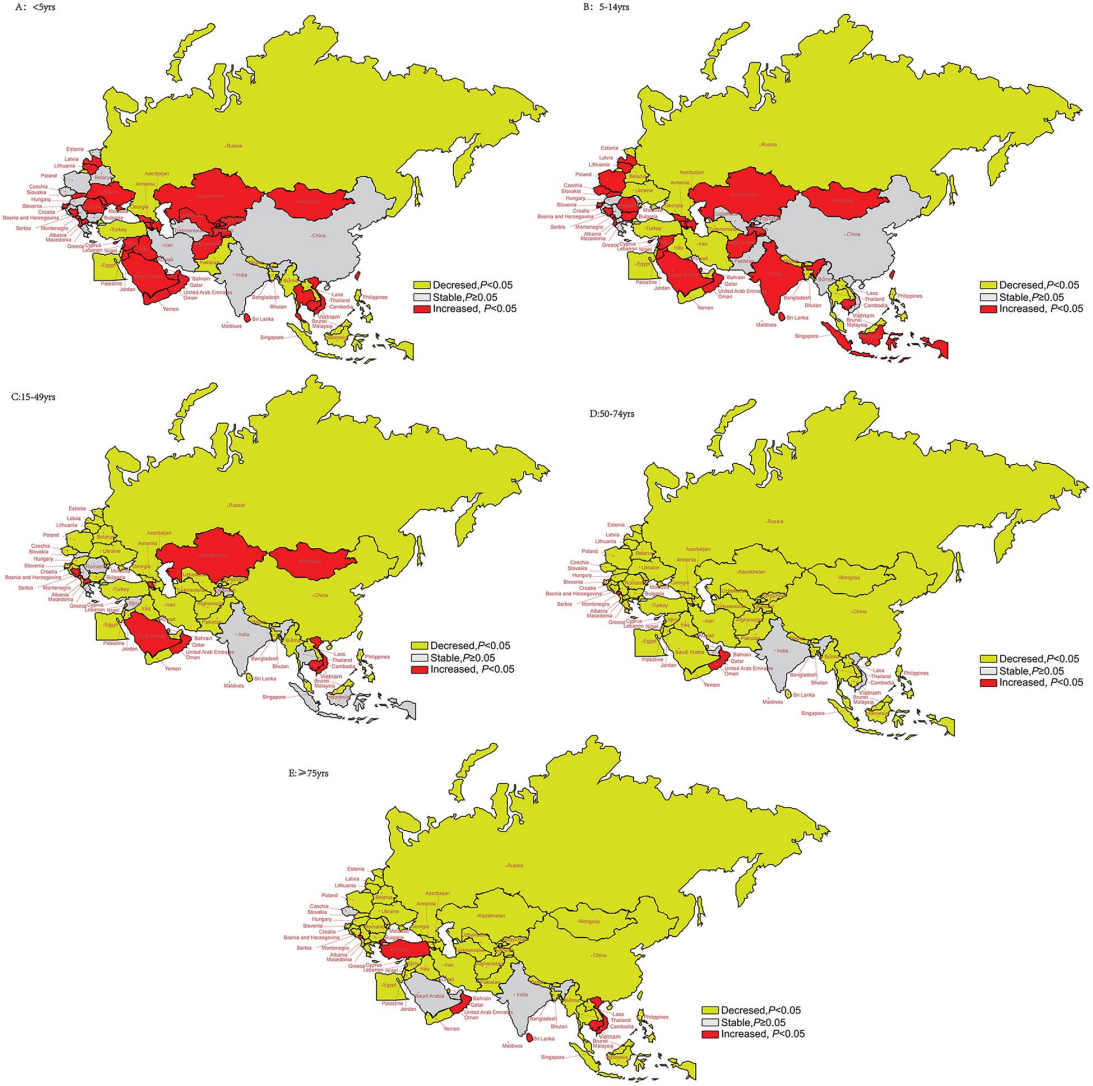
Trends of age-standardized YLDs rate stratified by age from 1990 to 2019 in “Belt & Road” countries. YLD rate in individuals aged <5 years; (B) YLD rate in individuals aged 5–14 years; (C) YLD rate in individuals aged 15–49 years; (D) YLD rate in individuals aged 50–74 years; (E) YLD rate in individuals aged ≥75 years.

Figure 5 and Table S6 illustrate the trends in age-standardized YLDs stratified by age groups in the B&R countries from 1990 to 2019. Age groups were categorized as <5 years, 5–14 years, 14–49 years, 50–74 years and ≥75 years. Many countries in Central Asia and Europe exhibited an increasing trend in YLDs for the <5-year age group. However, most countries experienced a downward trend in the 50–74- and ≥75-year age groups. Notably, Montenegro and Oman showed an increasing trend in age-standardized YLDs across all age groups. In China, the trend in age-standardized YLDs remained stable for the <5- and 5–14-year age groups but showed a downward trend in other age groups, with the lowest AAPC value observed in the 50–74-year age group: −2.64% (95% CI: −2.29% to −3.00%, *p* < .001).

### Risk factors and trends in age-standardized YLDs

**Figure 6.**
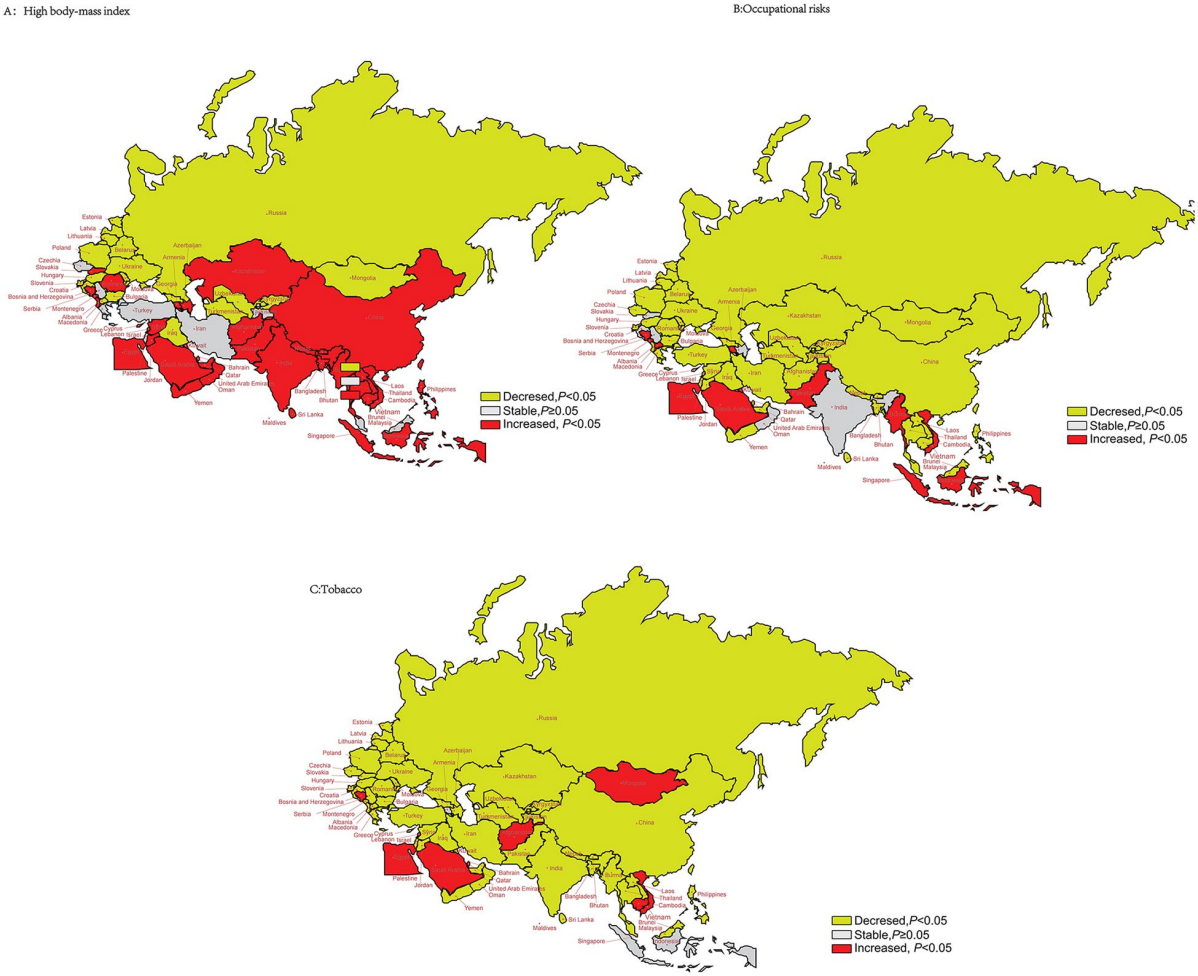
Trends regarding the age-standardized YLD rate contributed to risk factors from 1990 to 2019 in “Belt & Road” countries. (A) High body mass index; (B) occupational risks; (C) tobacco.

We also investigated the effects of high body mass index (BMI), occupational risk and tobacco use on the trend in age-standardized YLDs for asthma from 1990 to 2019 (Figure 6, Table S7). In East Asia, Central Asia, Southeast Asia (except Malaysia), North Africa and the Middle East (except Bahrain, Iran, Iraq, Palestine and Turkey), high BMI was related to a significant upward trend in age-standardized YLDs. Vietnam had the largest increase in the AAPC of age-standardized YLDs by 5.06% (95% CI: 4.83–5.29%, *p* < .001). In the B&R member countries, occupational risk and tobacco use mostly were related to a downward trend in YLDs. Among these three risk factors, the change in trends in age-standardized YLDs in Vietnam, Bosnia and Herzegovina, Montenegro, Egypt and Saudi Arabia increased significantly (*p* < .001).

## Discussion

The health status of the B&R member countries exhibits significant spatial heterogeneity. European countries generally boast of better health conditions than do countries in South Asia, Southeast Asia, Africa and the Middle East, which have poorer health conditions. Within African countries, medical resources are relatively scarce, making it challenging to effectively control health issues such as epidemics [7]. According to GBD 2015, there was a strong relationship between SDI and mortality [14]. Globally, a low mortality rate of asthma was observed in high SDI countries and a higher mortality rate was observed in low SDI countries in global. From 1990 to 2019, the mortality rate of asthma showed a decreasing trend in all SDI regions, especially from low SDI regions to high SDI regions, where the mortality rate of asthma decreased sequentially [5]. Under-diagnosis and inadequate treatment in low SDI regions led to more asthma deaths [15]. The incidence rate of asthma did not decrease with increasing SDI; rather, it was highest in regions with high SDI [5]. Regions characterized by high SDI demonstrate superior asthma monitoring and investigation systems, leading to a higher asthma detection rate. Conversely, regions with low SDI require additional support from medical resources.

YLDs measure the time people lose because of diseases that reduce their health but do not cause death. With respect to asthma, YLDs reflect the disease burden more effectively than incidence and mortality alone. There were significant differences in the trend in age-standardized YLDs among some B&R member countries between 1990 and 2019 and between 2010 and 2019. Taking China as an example, the AAPC age-standardized YLDs showed an upward trend from 2010 to 2019, whereas the opposite trend was observed between 1990 and 2019. This observation can be attributed to effective management measures and use of inhaled corticosteroids (ICSs) [16], as depicted in Figure S1. The 2006 report for the Global Initiative for Asthma introduced important new themes, emphasizing the goal of asthma treatment as achieving and maintaining clinical control [17]. In China, guidelines for the prevention and management of bronchial asthma were published in 2008, which may have contributed to the changes in YLDs during the specified period. The fact that ICSs have been demonstrated to improve all clinical manifestations of asthma and reduce asthma mortality is worth noting [18]. However, despite these advancements, asthma deaths still occur because of social deprivation and low adherence to ICS use, even in developed countries [19].

Globally, approximately half of the adult population with asthma consists of current or former tobacco smokers [20]. Smoking is often associated with poor clinical outcomes in asthma, including inadequate control and increased exacerbations [21], leading to airway remodelling, corticosteroid insensitivity and low-grade systemic inflammation. Among adults with asthma, LMICs have a higher proportion of smokers. WHO advocates measures for tobacco such as banning advertising, creating tobacco-free spaces, inserting health warnings on the packaging of tobacco products, and increased cigarette taxation. More than 71% of the world’s population were covered by tobacco cessation measure [22]. Between 1990 and 2015, the global prevalence of daily smoking fell significantly by 28.4% for men and 34.4% for women [23]. The B&R countries also actively respond to various smoking cessation measures. Since 1990, as one of the countries with the largest number of total smokers, China recorded significant decreases in male smoking prevalence suggesting sustained progress in tobacco control [23]. Effective tobacco control policies were ratified by the Philippines in 2005 [24]. Between 2005 and 2015, only Egypt recorded a significant increase in the smoking-attributable mortality rate among both sexes [23]. This could explain why our research shows that mostly countries show a decreasing trend in YLDs of asthma attributable to tobacco consumption. The goal of ‘Healthy China in 2030’ is to reduce the adult smoking rate from 27.7% in 2015 to 20% by 2030 [25]. Joint implementation of effective smoking cessation measures in China and all the B&R member countries is key to promoting a healthy B&R country.

There is evidence suggesting that obesity affects the risk of asthma [26,27]. Patients with asthma who are obese experience poor asthma control and higher healthcare utilization [28,29]. Obese adults with asthma often exhibit increased oxidative stress in the airways and corticosteroid insensitivity [29]. Fat accumulation in the abdomen and mediastinum can alter respiratory mechanisms, thereby altering the physiology and function of the lungs [30]. According to the GBD 2017, high BMI accounted for the largest number of asthma-related deaths since 2013 and has contributed the most to DALYs since 2003. Mortality resulting from high BMI is negatively correlated with SDI, resulting in a greater burden of asthma in regions with low SDI [13]. Considering the continuous increase in obesity prevalence, weight loss should be incorporated into the management of obese patients with asthma, particularly in East Asia, Central Asia, Southeast Asia, North Africa and the Middle East, according to our study.

Approximately, 10–25% of adult asthma cases are estimated to be caused by work-related exposure to allergens or irritants [31]. The mortality rate owing to occupational risk decreased and showed a negative association with SDI in the GBD 2017 [13]. In our study, the impact of occupational risk on the YLDs trend mostly showed a downward trend in the B&R member countries. However, attention is needed regarding the occupational risk of asthma in countries with low SDI. Evidence suggests that air pollution and nitrogen dioxide exposure negatively impact asthma outcomes, as they can worsen symptoms and reduce lung function [3]. While air quality in developed countries has been improving, it has worsened in developing countries. Severe air pollution affects people in these overpopulated regions, with 91% of the 4.2 million premature deaths in 2016 occurring in LMICs in Southeast Asia and Central Africa [32]. Minimizing exposure to indoor and outdoor pollutants can effectively improve asthma outcomes.

Furthermore, gender differences reflect the heterogeneity of asthma [33]. Factors such as sex hormones and genetic and epigenetic variations are important in causing sex differences. The incidence and severity of asthma in women increase after adulthood owing to hormonal fluctuations during puberty, the menstrual cycle, menopause and pregnancy [6]. Females are more susceptible to asthma and severe asthma than males [34]. Furthermore, there are more females than males with fewer atopic diseases, poorer corticosteroid responses and obese patients with steroid-refractory asthma [35]. From 1990 to 2017, the mortality rates and DALYs for chronic respiratory diseases decreased, with a more significant decline observed in males [13]. In the present study, the YLDs for males and females in most B&R member countries exhibited a decreasing trend. However, in some countries, males showed an upward trend in YLDs, while females showed the opposite trend. This suggests the need for increased attention to disease control in male patients.

This study had several limitations. First, the assessment of asthma incidence and mortality was limited by memory bias, access to medical services, and variations in self-reported health measurements. Second, the GBD had limitations, as the results were influenced by the quality of the population and disease data. Third, this study used data from 1990 to 2019 owing to the lack of more recent data.

In conclusion, this study revealed a general downward trend in the burden of asthma from 1990 to 2019 in the B&R member countries. However, there was an upward trend in the AAPC age-standardized YLDs for asthma from 2010 to 2019 in some East Asian, Central Asian, North African and Middle Eastern countries; this contradicts the overall downward trend observed from 1990 to 2019. Furthermore, a consistent decrease was observed when analysing the trend of YLDs among males and females in most B&R member countries. However, it is worth noting that a high BMI was associated with an increased trend in YLDs. Therefore, paying more attention to the relationship between high BMI and disease burden is crucial.

## Supplementary Material

Supplemental Material

## Data Availability

The data that support the findings of this study are available in Global Health Data Exchange GBD 2019 website at https://vizhub.healthdata.org/gbd-results/.
