# Supplementary material for: Trends in disease burden and risk factors of asthma from 1990 to 2019 in Belt and Road Initiative countries: evidence from the Global Burden of Disease Study 2019
Source: Ann Med. 2024 Sep 6;56(1):2399964. doi: 10.1080/07853890.2024.2399964 (PMC11382694; doi:10.1080/07853890.2024.2399964)
Supplement: Supplemental Material [file IANN_A_2399964_SM5095.zip › Supp_data/STables.docx]

**Table S1 The absolute number of incidence, mortality, prevanlence, YLDs, YLLs and DALYs due to asthma in 2019.**

|  | **Incidence** | | **Mortality** | | **Prevanlence** | | **YLDs** | | **YLLs** | | **DALYs** | |
| --- | --- | --- | --- | --- | --- | --- | --- | --- | --- | --- | --- | --- |
| **Countries** | **Number** | ***95%UI*** | **Number** | ***95%UI*** | **Number** | ***95%UI*** | **Number** | ***95%UI*** | **Number** | ***95%UI*** | **Number** | ***95%UI*** |
| High SDI | 7056076.39 | 5900059.47 - 8406845.70 | 17959.47 | 15624.44 - 20181.21 | 68952653.03 | 60703693.89-78756180.52 | 2645686.52 | 1737984.08 - 3845124.48 | 356539.97 | 325096.79 - 393756.80 | 3002226.50 | 2091655.03 - 4202058.25 |
| High-middle SDI | 5410537.16 | 4349432.95-6726612.71 | 35675.00 | 30692.13-40377.47 | 42846686.13 | 35939860.07-51183980.04 | 1662846.06 | 1084433.17-2474001.14 | 691080.36 | 609403.83-782012.22 | 2353926.42 | 1761731.68-3168080.03 |
| Middle SDI | 10369656.21 | 8174205.72 - 13107056.63 | 116849.67 | 100287.16 - 131917.76 | 67251100.08 | 56236270.71-81268922.84 | 2635538.10 | 1708971.00 - 3945782.97 | 2793940.26 | 2422954.30 - 3154946.56 | 5429478.36 | 4363001.26 - 6758389.53 |
| Low-middle SDI | 7096393.73 | 5718775.64-8795880.83 | 200059.96 | 143311.89-257378.13 | 45235059.97 | 38351484.44-54097332.44 | 1753886.12 | 1153116.87-2593812.68 | 4804346.40 | 3577612.06-5936221.84 | 6558232.52 | 5191112.20-7974146.75 |
| Low SDI | 7016215.56 | 5410570.07 - 9113991.52 | 90179.54 | 65746.07 - 127842.03 | 37911430.07 | 31439352.88-46543193.70 | 1490133.24 | 953066.03 - 2217090.57 | 2698524.91 | 2091460.80 - 3537523.08 | 4188658.15 | 3320169.84 - 5258768.30 |
| **East Asia** | | | | | | | | | | | | |
| China | 3761276.75 | 2901080.04 - 4917885.65 | 24750.22 | 20244.86 - 30769.13 | 24766952.29 | 20076066.34-30666151.01 | 971497.66 | 615814.51 - 1472285.20 | 441653.91 | 360272.27 - 552909.48 | 1413151.57 | 1052300.51 - 1918822.31 |
| **Central Asia** | | | | | | | | | | | | |
| Armenia | 9170.24 | 6954.04 - 11748.35 | 17.97 | 13.80 - 23.04 | 62215.66 | 46975.13-76441.97 | 2432.54 | 1497.74 - 3661.35 | 349.84 | 268.85 - 446.26 | 2782.38 | 1850.05 - 3988.34 |
| Azerbaijan | 31119.55 | 24184.60 - 39603.27 | 364.22 | 259.40 - 591.47 | 189910.21 | 156275.45-234270.89 | 7475.39 | 4757.33 - 11252.57 | 8205.93 | 6057.20 - 13164.59 | 15681.32 | 11998.15 - 21013.47 |
| Georgia | 10669.43 | 8371.29 - 13594.77 | 115.97 | 90.85 - 156.14 | 69581.84 | 56805.01-85459.17 | 2708.33 | 1704.47 - 4057.05 | 2394.15 | 1930.58 - 3009.83 | 5102.48 | 3991.04 - 6522.09 |
| Kazakhstan | 51667.43 | 39763.40 - 66585.74 | 1259.48 | 928.99 - 1611.59 | 286976.47 | 232366.60-356099.44 | 11260.11 | 7164.56 - 17228.68 | 26362.65 | 20164.39 - 33278.10 | 37622.77 | 29570.97 - 46982.29 |
| Kyrgyzstan | 27312.45 | 20367.10 - 36254.02 | 57.06 | 45.94 - 80.59 | 156012.09 | 124978.14-197290.78 | 6170.11 | 3889.99 - 9435.37 | 1574.36 | 1238.99 - 2336.07 | 7744.47 | 5436.23 - 11033.54 |
| Mongolia | 12166.85 | 9208.78 - 16024.21 | 105.03 | 77.55 - 154.20 | 67113.67 | 55071.32-82906.07 | 2641.29 | 1677.50 - 3973.46 | 2471.12 | 1799.89 - 3704.89 | 5112.41 | 3851.06 - 6786.67 |
| Tajikistan | 37083.63 | 27174.44 - 49748.48 | 250.06 | 191.44 - 327.13 | 194584.32 | 154929.70-248712.27 | 7699.50 | 4856.42 - 11857.38 | 5062.80 | 3880.00 - 6858.68 | 12762.30 | 9476.45 - 17027.91 |
| Turkmenistan | 17965.33 | 13165.31 - 24196.91 | 49.24 | 32.96 - 92.56 | 101613.78 | 78998.91-129040.41 | 4024.71 | 2451.12 - 6182.00 | 1511.56 | 1034.59 - 2601.44 | 5536.26 | 3855.40 - 7773.96 |
| Uzbekistan | 156519.05 | 121360.71 - 202861.36 | 1123.06 | 885.57 - 1640.88 | 944061.85 | 779576.43-1162862.71 | 37089.61 | 23819.30 - 55987.02 | 31306.51 | 24604.95 - 44029.04 | 68396.13 | 52527.00 - 88502.42 |
| **South Asia** | | | | | | | | | | | |  |
| Bangladesh | 312711.07 | 266090.97 - 366796.23 | 10719.29 | 7475.33 - 17539.04 | 2073478.31 | 1801990.97-2362799.75 | 79825.89 | 52818.44 - 114684.40 | 256755.48 | 181214.68 - 411494.56 | 336581.37 | 253568.70 - 491855.92 |
| Bhutan | 1482.72 | 1276.24 - 1751.51 | 75.28 | 52.48 - 112.69 | 9763.37 | 8608.13-11051.01 | 375.81 | 245.28 - 543.38 | 1628.41 | 1091.99 - 2447.68 | 2004.22 | 1450.40 - 2870.85 |
| India | 4533397.76 | 3732737.34 - 5478017.92 | 198798.87 | 129621.75 - 271915.87 | 34305973.24 | 27950714.40-41323723.48 | 1305163.77 | 839030.54 - 1930247.47 | 4533600.98 | 3082605.36 - 5949567.82 | 5838764.76 | 4244934.47 - 7429627.78 |
| Nepal | 48737.05 | 41232.54 - 57615.70 | 6900.81 | 4301.41 - 9792.62 | 289922.63 | 249850.52-332070.17 | 11154.23 | 7322.51 - 16464.85 | 154352.51 | 98282.40 - 217049.70 | 165506.73 | 108362.74 - 230017.67 |
| Pakistan | 541632.60 | 435288.27 - 685683.82 | 15693.77 | 12142.76 - 21425.53 | 3192551.52 | 2659807.00-3848337.33 | 124153.29 | 80079.03 - 182789.58 | 440576.00 | 347874.20 - 569451.33 | 564729.30 | 453492.27 - 709475.38 |
| **Southeast Asia** | | | | | | | | | | | | |
| Cambodia | 69235.40 | 54268.49 - 89010.56 | 1359.33 | 1038.87 - 1674.89 | 386095.49 | 322587.17-474534.85 | 15193.58 | 9754.34 - 22902.83 | 33751.09 | 25906.23 - 41528.23 | 48944.67 | 39096.15 - 60141.74 |
| Indonesia | 1309435.06 | 1067022.46 - 1619266.52 | 26457.66 | 21282.11 - 32819.96 | 8673884.37 | 7367856.06-10270736.17 | 340822.93 | 222112.87 - 496943.38 | 704161.08 | 566393.14 - 866577.96 | 1044984.01 | 853095.56 - 1263065.26 |
| Lao | 26664.10 | 21625.61 - 33304.04 | 820.92 | 590.79 - 1126.27 | 147308.67 | 126745.94-175671.30 | 5812.42 | 3685.42 - 8615.87 | 24366.85 | 17294.64 - 32842.07 | 30179.27 | 22655.32 - 38816.88 |
| Malaysia | 125335.66 | 100250.58 - 162124.34 | 938.72 | 680.79 - 1411.60 | 792596.05 | 662894.81-969016.49 | 31239.96 | 20151.91 - 46768.95 | 25678.78 | 19028.93 - 36282.74 | 56918.74 | 43122.10 - 73572.14 |
| Maldives | 1801.53 | 1385.11 - 2324.64 | 10.39 | 8.30 - 12.94 | 11017.60 | 8811.18-13954.64 | 437.13 | 270.76 - 665.04 | 223.10 | 180.88 - 273.85 | 660.23 | 485.32 - 900.22 |
| Burma | 187023.67 | 158583.95 - 224645.55 | 11043.03 | 7905.19 - 13819.90 | 1065997.14 | 933365.50-1212653.04 | 41613.63 | 27311.74 - 60510.32 | 272037.47 | 200082.90 - 346375.98 | 313651.10 | 238290.79 - 390682.02 |
| Philippines | 880783.21 | 690740.44 - 1142588.59 | 11776.38 | 9712.04 - 14703.12 | 5218947.60 | 4372997.03-6402826.38 | 206681.08 | 132732.97 - 308100.19 | 369991.81 | 309654.17 - 433655.19 | 576672.89 | 479743.04 - 693345.76 |
| Sri Lanka | 119666.34 | 102098.06 - 140633.92 | 8228.72 | 5744.13 - 10722.70 | 799525.86 | 705681.51-914751.36 | 30481.28 | 20106.74 - 44301.47 | 136443.73 | 95549.29 - 180192.63 | 166925.00 | 124625.40 - 211782.18 |
| Thailand | 294342.70 | 245693.62 - 351331.98 | 4193.72 | 3053.05 - 5581.75 | 2309630.86 | 2005711.93-2688542.41 | 89856.86 | 58587.31 - 134199.29 | 82068.60 | 59491.61 - 110119.06 | 171925.45 | 131778.60 - 222440.89 |
| Viet Nam | 419716.48 | 345208.88 - 519093.75 | 6834.50 | 2814.45 - 9199.92 | 2849226.88 | 2429245.31-3358420.30 | 111383.41 | 72971.10 - 165959.12 | 142821.60 | 60589.28 - 194014.91 | 254205.01 | 164896.32 - 324341.73 |
| **High-income Asia pacific** | | | | | | | | | | | | |
| Brunei | 2224.60 | 1793.64 - 2740.65 | 14.45 | 11.92 - 17.45 | 17726.41 | 14630.67-21813.50 | 690.84 | 436.96 - 1046.65 | 275.22 | 230.19 - 333.60 | 966.06 | 719.35 - 1315.13 |
| Singapore | 20939.73 | 17119.12 - 25926.80 | 45.10 | 36.94 - 67.19 | 199395.45 | 165088.97-242228.77 | 7801.50 | 4980.06 - 11725.01 | 1036.20 | 845.60 - 1598.00 | 8837.69 | 5943.03 - 12756.96 |
| **North Africa and Middle East** | | | | | | | | | | | | |
| Afghanistan | 281630.54 | 217451.17 - 363785.35 | 2947.18 | 1953.88 - 4134.08 | 1515531.73 | 1250583.77-1877887.06 | 59493.24 | 37750.45 - 90816.57 | 93652.00 | 63400.21 - 130682.46 | 153145.24 | 115193.32 - 198998.79 |
| Bahrain | 6166.11 | 4903.95 - 7613.73 | 31.11 | 23.81 - 41.79 | 46332.60 | 38382.26-56216.15 | 1816.74 | 1141.32 - 2712.42 | 920.63 | 700.05 - 1263.24 | 2737.36 | 2048.08 - 3699.18 |
| Egypt | 566302.76 | 441856.43 - 720245.06 | 7489.93 | 5033.26 - 10490.67 | 3347076.34 | 2743235.62-4121161.60 | 132045.70 | 83688.83 - 197114.31 | 197708.26 | 135306.32 - 281780.04 | 329753.96 | 248868.56 - 426178.52 |
| Iran | 415327.62 | 327426.02 - 524205.25 | 3605.61 | 3095.35 - 4029.75 | 2635572.90 | 2182430.09-3205593.56 | 103085.90 | 66253.12 - 155079.49 | 75646.74 | 67236.22 - 86658.73 | 178732.64 | 141138.90 - 230621.06 |
| Iraq | 248615.49 | 190297.30 - 326435.68 | 1157.34 | 891.31 - 1474.27 | 1451875.53 | 1169492.13-1834319.03 | 57370.48 | 36350.46 - 87177.28 | 31502.43 | 23665.56 - 41319.13 | 88872.91 | 65705.60 - 119964.95 |
| Jordan | 76041.97 | 57524.96 - 98882.73 | 142.99 | 115.64 - 178.41 | 470579.07 | 373464.89-600637.68 | 18681.95 | 11560.05 - 28593.52 | 3987.91 | 3255.38 - 4956.46 | 22669.86 | 15490.98 - 32674.20 |
| Kuwait | 23468.83 | 18101.53 - 29633.14 | 32.93 | 26.27 - 41.62 | 174108.87 | 138549.35-221830.84 | 6862.11 | 4241.82 - 10301.65 | 910.93 | 722.76 - 1190.94 | 7773.04 | 5137.61 - 11224.94 |
| Lebanon | 33802.18 | 26856.99 - 42066.93 | 278.79 | 177.05 - 376.37 | 227323.47 | 186844.61-274255.63 | 8859.34 | 5645.73 - 13276.70 | 5423.18 | 3566.35 - 7390.10 | 14282.53 | 10619.64 - 18865.07 |
| Oman | 25413.40 | 19974.75 - 32468.56 | 6.99 | 5.60 - 9.07 | 172074.98 | 136810.21-218228.73 | 6803.70 | 4241.61 - 10429.10 | 200.02 | 161.02 - 259.56 | 7003.72 | 4432.61 - 10629.14 |
| Palestine | 30541.39 | 22825.39 - 40046.08 | 98.89 | 76.30 - 120.38 | 167758.89 | 130859.35-216934.67 | 6644.88 | 4146.23 - 10256.89 | 2433.79 | 1925.18 - 2978.69 | 9078.67 | 6532.04 - 12744.01 |
| Qatar | 11543.76 | 9155.66 - 14313.41 | 8.26 | 5.96 - 12.18 | 84800.06 | 67145.66-108244.68 | 3347.60 | 2116.36 - 5066.63 | 336.06 | 242.94 - 486.36 | 3683.66 | 2461.20 - 5454.19 |
| Saudi Arabia | 132867.08 | 105712.78 - 165421.45 | 906.22 | 684.12 - 1225.22 | 853996.12 | 705952.38-1027815.08 | 33757.87 | 21556.01 - 49243.10 | 29130.46 | 21323.80 - 40282.58 | 62888.32 | 48236.93 - 81569.99 |
| Syrian Arab Republic | 76301.37 | 60880.27 - 93829.05 | 1171.84 | 873.94 - 1539.10 | 469398.58 | 397788.76-559452.72 | 18365.39 | 11763.13 - 26947.16 | 31194.92 | 23155.06 - 41453.47 | 49560.31 | 39101.51 - 62444.09 |
| Turkey | 515559.02 | 435559.33 - 611835.64 | 2666.19 | 1981.85 - 3587.67 | 4082369.93 | 3517473.53-4743626.70 | 157498.23 | 102000.72 - 229711.91 | 46452.37 | 35743.45 - 61304.95 | 203950.60 | 147952.50 - 277542.50 |
| United Arab Emirates | 62279.16 | 50671.29 - 76300.65 | 550.09 | 312.57 - 1011.53 | 614690.18 | 514497.66-719007.06 | 24094.04 | 15391.97 - 35699.55 | 22273.18 | 12418.69 - 41063.43 | 46367.22 | 32308.95 - 66889.33 |
| Yemen | 214370.77 | 161769.54 - 277934.07 | 2124.65 | 1546.72 - 3038.78 | 1189810.06 | 968154.28-1487502.54 | 46736.28 | 29465.75 - 71274.96 | 60415.79 | 43852.98 - 84442.06 | 107152.07 | 82283.42 - 139677.23 |
| **Central Europe** | | | | | | | | | | | | |
| Albania | 11128.20 | 9289.33 - 13442.61 | 143.29 | 99.36 - 199.28 | 79785.19 | 66536.61-96602.43 | 3084.99 | 1961.99 - 4589.12 | 2291.19 | 1575.93 - 3187.76 | 5376.18 | 3990.03 - 6982.38 |
| Bosnia and Herzegovina | 18566.72 | 15591.41 - 22068.73 | 56.91 | 42.89 - 73.91 | 161181.22 | 134157.15-192943.12 | 6153.25 | 3915.58 - 9060.29 | 921.09 | 687.28 - 1196.72 | 7074.34 | 4809.23 - 9973.71 |
| Bulgaria | 29693.27 | 24390.89 - 35436.93 | 51.81 | 38.36 - 67.70 | 260802.85 | 213949.07-311776.07 | 10037.13 | 6413.90 - 14923.96 | 1001.45 | 743.73 - 1304.64 | 11038.58 | 7404.34 - 15928.09 |
| Croatia | 21660.73 | 18387.72 - 25716.81 | 55.17 | 42.17 - 70.33 | 188078.24 | 158810.02-223334.43 | 7165.07 | 4639.93 - 10482.87 | 815.68 | 621.73 - 1044.13 | 7980.75 | 5381.39 - 11339.07 |
| Czechia | 40126.64 | 32773.69 - 48307.11 | 116.78 | 88.98 - 146.22 | 327052.30 | 269708.16-392255.67 | 12576.54 | 7963.58 - 18479.72 | 2364.21 | 1806.71 - 3028.82 | 14940.76 | 10280.79 - 20862.08 |
| Hungary | 37786.14 | 31130.13 - 45480.75 | 109.68 | 85.35 - 137.41 | 321229.13 | 265613.64-385389.84 | 12401.03 | 7961.44 - 18302.59 | 2225.52 | 1733.63 - 2776.43 | 14626.55 | 10149.06 - 20478.79 |
| Montenegro | 2844.02 | 2285.19 - 3541.95 | 3.10 | 2.32 - 4.01 | 22895.84 | 18278.45-28178.49 | 887.21 | 556.29 - 1329.96 | 59.11 | 44.80 - 76.97 | 946.32 | 616.34 - 1392.79 |
| Macedonia | 14379.33 | 12304.17 - 16608.08 | 57.83 | 43.59 - 74.03 | 123319.78 | 105483.46-145172.12 | 4709.05 | 3057.99 - 6977.90 | 1006.73 | 752.32 - 1304.29 | 5715.78 | 4058.85 - 7895.55 |
| Poland | 248709.78 | 204209.48 - 303392.67 | 523.58 | 427.94 - 690.63 | 2214693.36 | 1848569.88-2654560.39 | 85210.63 | 54871.45 - 125750.29 | 9454.41 | 7789.23 - 12085.41 | 94665.03 | 64841.94 - 135619.43 |
| Romania | 96468.23 | 78600.61 - 118130.70 | 254.36 | 199.39 - 328.77 | 844316.44 | 694481.94-1015130.73 | 32564.72 | 20672.34 - 48414.96 | 4249.77 | 3320.06 - 5523.13 | 36814.49 | 25024.20 - 52756.51 |
| Serbia | 34347.15 | 28904.02 - 40784.24 | 331.38 | 256.63 - 416.69 | 260376.53 | 224079.91-303470.99 | 10011.11 | 6508.82 - 14731.81 | 5671.69 | 4387.05 - 7303.10 | 15682.80 | 11885.90 - 20507.63 |
| Slovakia | 19980.92 | 16346.04 - 24301.14 | 52.03 | 39.38 - 70.77 | 159787.45 | 131166.69-192791.58 | 6182.38 | 3894.81 - 9051.31 | 1042.42 | 794.23 - 1416.23 | 7224.80 | 4955.97 - 10216.85 |
| Slovenia | 11279.04 | 9384.70 - 13554.85 | 19.86 | 14.98 - 26.51 | 102604.13 | 84761.84-123241.22 | 3924.52 | 2514.11 - 5842.04 | 316.66 | 240.82 - 424.21 | 4241.18 | 2796.83 - 6139.36 |
| **Eastern Europe** | | | | | | | | | | | | |
| Belarus | 42427.50 | 34444.09 - 52939.61 | 126.83 | 79.61 - 287.31 | 328033.37 | 271029.94-396927.20 | 12705.67 | 8172.97 - 18816.46 | 2661.03 | 1644.73 - 6025.79 | 15366.70 | 10345.16 - 21798.16 |
| Es-nia | 3678.51 | 2963.40 - 4522.68 | 29.59 | 22.02 - 40.58 | 26128.41 | 21890.75-30890.36 | 1012.58 | 659.69 - 1477.92 | 523.92 | 390.50 - 715.23 | 1536.50 | 1145.15 - 2025.57 |
| Latvia | 7692.31 | 6238.11 - 9424.29 | 35.27 | 26.77 - 53.87 | 60696.29 | 51538.88-72357.78 | 2340.63 | 1526.10 - 3407.26 | 656.63 | 494.28 - 996.32 | 2997.26 | 2123.55 - 4115.35 |
| Lithuania | 9590.71 | 7694.92 - 11730.83 | 36.75 | 28.17 - 51.91 | 73987.00 | 61789.49-88188.76 | 2855.11 | 1847.40 - 4192.18 | 705.62 | 537.77 - 1000.17 | 3560.73 | 2534.91 - 4922.39 |
| Moldova | 12856.72 | 10116.86 - 16199.51 | 30.60 | 24.64 - 41.29 | 98058.45 | 78492.61-119716.47 | 3813.83 | 2368.46 - 5709.15 | 670.32 | 530.78 - 937.65 | 4484.15 | 3061.17 - 6340.27 |
| Russian Federation | 507708.60 | 393295.11 - 649531.93 | 1786.37 | 1498.89 - 2443.28 | 3507708.44 | 2866449.67-4254388.34 | 136372.67 | 87496.43 - 202750.89 | 40288.09 | 33613.05 - 54977.13 | 176660.75 | 126447.21 - 243569.86 |
| Ukraine | 171318.36 | 130592.90 - 221689.21 | 280.12 | 211.17 - 521.46 | 1268398.99 | 1019017.37-1553151.05 | 49499.20 | 31615.38 - 74293.75 | 6659.97 | 5052.25 - 12247.30 | 56159.17 | 37710.78 - 81008.82 |
| **Western Europe** | | | | | | | | | | | | |
| Cyprus | 5903.99 | 4824.48 - 7170.04 | 29.41 | 21.08 - 43.17 | 99524.74 | 80592.50-120953.32 | 3839.10 | 2432.99 - 5740.68 | 397.99 | 308.81 - 527.99 | 4237.09 | 2832.16 - 6127.03 |
| Greece | 36457.52 | 29721.54 - 43717.77 | 66.95 | 50.69 - 81.61 | 591070.09 | 487643.67-712681.18 | 22692.19 | 14490.69 - 33766.87 | 1091.22 | 897.39 - 1283.78 | 23783.40 | 15568.50 - 34956.48 |
| Israel | 40691.13 | 31597.17 - 50555.01 | 134.08 | 110.36 - 178.28 | 465362.11 | 392157.35-560172.17 | 18159.06 | 11636.08 - 27085.47 | 2409.42 | 2064.95 - 3178.94 | 20568.48 | 14044.23 - 29635.28 |

**Table S2 The age-standardized rates of incidence, mortality, prevanlence, YLLs, YLDs and DALYs in 1990 and 2019 in “the Belt & Road” countries.**

|  | **1990(/100,000)** | | | | | | **2019(/100,000)** | | | | | |
| --- | --- | --- | --- | --- | --- | --- | --- | --- | --- | --- | --- | --- |
| **Countries** | **Mortality** | **Incidence** | **Prevalence** | **YLDS** | **YLLs** | **DALYs** | **Mortality** | **Incidence** | **Prevalence** | **YLDS** | **YLLs** | **DALYs** |
| **Global** | 11.91 | 580.09 | 4496.93 | 173.62 | 302.66 | 476.28 | 5.80 | 504.28 | 3415.53 | 132.99 | 140.65 | 273.63 |
| High SDI | 3.57 | 933.66 | 8329.46 | 323.07 | 86.18 | 409.25 | 0.96 | 897.36 | 6855.06 | 267.93 | 25.76 | 293.69 |
| High-middle SDI | 5.50 | 559.79 | 4240.89 | 164.17 | 122.62 | 286.79 | 1.83 | 474.27 | 3103.02 | 121.82 | 36.51 | 158.33 |
| Middle SDI | 13.26 | 523.33 | 3268.82 | 127.58 | 292.36 | 419.94 | 5.33 | 483.85 | 2927.13 | 114.97 | 115.67 | 230.64 |
| Low-middle SDI | 32.29 | 475.36 | 3154.12 | 120.94 | 738.49 | 859.43 | 16.29 | 415.97 | 2713.25 | 104.40 | 341.50 | 445.90 |
| Low SDI | 33.26 | 594.50 | 4007.06 | 154.27 | 804.43 | 958.70 | 18.94 | 544.09 | 3468.63 | 134.11 | 419.79 | 553.90 |
| **East Asia** | | | | | | | | | | | | |
| China | 6.37 | 394.58 | 2296.63 | 90.17 | 119.07 | 209.24 | 1.51 | 355.33 | 1974.16 | 78.31 | 24.50 | 102.81 |
| **Central Asia** | | | | | | | | | | | | |
| Armenia | 2.41 | 333.69 | 1951.76 | 76.36 | 50.79 | 127.15 | 0.45 | 352.22 | 2093.13 | 82.48 | 8.61 | 91.09 |
| Azerbaijan | 9.67 | 351.95 | 2114.05 | 82.76 | 202.57 | 285.33 | 5.34 | 343.45 | 1961.21 | 77.14 | 95.24 | 172.37 |
| Georgia | 6.65 | 343.46 | 2093.30 | 82.16 | 153.88 | 236.04 | 1.94 | 342.53 | 1958.92 | 77.20 | 48.13 | 125.32 |
| Kazakhstan | 11.44 | 279.75 | 1600.12 | 62.24 | 239.37 | 301.61 | 8.44 | 285.86 | 1590.71 | 62.35 | 158.65 | 221.01 |
| Kyrgyzstan | 5.13 | 476.68 | 3441.90 | 133.79 | 129.69 | 263.48 | 1.32 | 394.33 | 2417.20 | 95.10 | 31.25 | 126.35 |
| Mongolia | 16.78 | 408.26 | 2609.96 | 100.70 | 325.78 | 426.48 | 6.31 | 361.35 | 2114.21 | 82.64 | 114.59 | 197.24 |
| Tajikistan | 14.15 | 407.07 | 2610.93 | 101.13 | 246.01 | 347.14 | 9.39 | 385.31 | 2264.74 | 88.30 | 135.97 | 224.27 |
| Turkmenistan | 10.64 | 432.38 | 2936.57 | 114.83 | 269.40 | 384.23 | 1.31 | 344.47 | 2011.28 | 79.41 | 34.24 | 113.65 |
| Uzbekistan | 12.57 | 560.81 | 4267.92 | 164.97 | 281.97 | 446.94 | 7.22 | 469.12 | 3058.75 | 119.00 | 146.51 | 265.51 |
| **South Asia** | | | | | | | | | | | | |
| Bangladesh | 32.58 | 266.77 | 1855.79 | 70.72 | 799.89 | 870.61 | 8.83 | 207.54 | 1390.91 | 53.29 | 190.11 | 243.40 |
| Bhutan | 34.27 | 291.80 | 2022.18 | 77.09 | 762.45 | 839.55 | 15.23 | 215.15 | 1429.78 | 54.64 | 287.18 | 341.82 |
| India | 41.98 | 419.33 | 2970.59 | 111.50 | 865.67 | 977.17 | 20.05 | 356.64 | 2680.88 | 101.21 | 397.31 | 498.52 |
| Nepal | 63.26 | 204.91 | 1254.04 | 47.61 | 1381.92 | 1429.52 | 36.40 | 175.47 | 1072.46 | 40.89 | 700.11 | 740.99 |
| Pakistan | 28.09 | 309.23 | 2133.26 | 81.44 | 618.92 | 700.36 | 16.39 | 252.16 | 1702.08 | 65.01 | 349.77 | 414.78 |
| **Southeast Asia** | | | | | | | | | | | | |
| Cambodia | 26.22 | 375.03 | 2172.79 | 84.35 | 612.22 | 696.57 | 13.67 | 414.91 | 2403.29 | 93.90 | 274.50 | 368.41 |
| Indonesia | 26.58 | 583.45 | 3811.87 | 147.93 | 649.69 | 797.62 | 14.87 | 544.94 | 3431.25 | 134.41 | 321.21 | 455.62 |
| Lao | 53.40 | 417.34 | 2401.57 | 93.91 | 1460.34 | 1554.24 | 21.11 | 368.90 | 2140.92 | 83.77 | 478.21 | 561.99 |
| Malaysia | 16.75 | 480.71 | 3348.11 | 129.86 | 401.18 | 531.04 | 3.83 | 433.51 | 2624.70 | 103.37 | 89.55 | 192.92 |
| Maldives | 23.37 | 595.28 | 3624.40 | 141.64 | 544.95 | 686.58 | 4.20 | 421.60 | 2422.72 | 95.82 | 71.63 | 167.46 |
| Burma | 74.11 | 384.93 | 2178.29 | 84.41 | 1775.38 | 1859.79 | 27.80 | 358.26 | 2016.24 | 78.39 | 586.09 | 664.49 |
| Philippines | 32.01 | 904.92 | 5870.10 | 230.16 | 759.44 | 989.60 | 16.36 | 742.69 | 4628.31 | 182.20 | 406.80 | 589.00 |
| Sri Lanka | 80.95 | 541.33 | 3611.24 | 138.89 | 1385.63 | 1524.52 | 40.31 | 563.80 | 3541.69 | 135.56 | 591.38 | 726.95 |
| Thailand | 15.02 | 561.66 | 3917.91 | 151.75 | 318.09 | 469.84 | 4.41 | 535.35 | 3411.53 | 134.36 | 91.21 | 225.57 |
| Viet Nam | 20.56 | 422.53 | 2603.15 | 101.50 | 406.74 | 508.24 | 8.68 | 485.57 | 3092.64 | 120.88 | 161.39 | 282.28 |
| **High-income Asia pacific** | | | | | | | | | | | | |
| Brunei | 30.45 | 669.84 | 5638.87 | 214.87 | 466.56 | 681.43 | 10.94 | 622.60 | 4887.06 | 187.66 | 139.82 | 327.48 |
| Singapore | 5.62 | 693.90 | 6704.47 | 260.48 | 155.36 | 415.84 | 0.62 | 500.44 | 3667.15 | 144.91 | 14.14 | 159.05 |
| **North Africa and Middle East** | | | | | | | | | | | | |
| Afghanistan | 45.35 | 652.33 | 4740.10 | 180.81 | 1065.82 | 1246.63 | 25.78 | 631.24 | 4281.02 | 164.12 | 565.14 | 729.26 |
| Bahrain | 14.96 | 656.32 | 4485.51 | 174.00 | 322.96 | 496.96 | 4.87 | 586.34 | 3639.21 | 142.46 | 91.69 | 234.15 |
| Egypt | 28.54 | 584.67 | 3697.98 | 144.36 | 681.61 | 825.97 | 13.66 | 554.85 | 3486.80 | 136.34 | 291.25 | 427.60 |
| Iran | 18.52 | 646.00 | 4235.23 | 163.92 | 365.39 | 529.31 | 5.61 | 544.09 | 3280.14 | 128.16 | 104.11 | 232.27 |
| Iraq | 14.93 | 696.37 | 5006.80 | 193.69 | 349.53 | 543.22 | 5.71 | 555.57 | 3446.54 | 134.97 | 120.97 | 255.94 |
| Jordan | 9.28 | 631.38 | 4273.34 | 167.10 | 202.04 | 369.14 | 2.61 | 629.02 | 4044.69 | 159.33 | 53.00 | 212.33 |
| Kuwait | 5.57 | 659.01 | 4470.85 | 175.49 | 124.03 | 299.52 | 1.46 | 641.21 | 4174.46 | 164.44 | 29.16 | 193.60 |
| Lebanon | 15.24 | 661.01 | 4514.71 | 175.57 | 299.86 | 475.43 | 5.45 | 671.71 | 4443.27 | 173.34 | 103.97 | 277.31 |
| Oman | 1.87 | 480.51 | 3084.05 | 120.78 | 39.50 | 160.28 | 0.64 | 616.57 | 4053.95 | 158.96 | 10.98 | 169.94 |
| Palestine | 13.09 | 558.87 | 3654.66 | 141.91 | 271.74 | 413.65 | 5.21 | 547.77 | 3332.38 | 130.24 | 96.98 | 227.22 |
| Qatar | 4.66 | 551.73 | 3410.60 | 133.72 | 101.32 | 235.04 | 1.36 | 554.69 | 3460.53 | 135.63 | 25.27 | 160.90 |
| Saudi Arabia | 15.94 | 373.60 | 2092.99 | 81.99 | 334.21 | 416.20 | 6.15 | 461.34 | 2642.17 | 104.06 | 124.23 | 228.29 |
| Syrian Arab Republic | 22.36 | 540.62 | 3348.28 | 130.73 | 496.26 | 627.00 | 12.38 | 544.30 | 3268.89 | 127.41 | 255.74 | 383.15 |
| Turkey | 9.83 | 741.21 | 5503.39 | 213.35 | 221.82 | 435.17 | 3.32 | 718.96 | 5102.54 | 197.58 | 55.90 | 253.49 |
| United Arab Emirates | 33.65 | 941.45 | 8100.42 | 313.94 | 747.83 | 1061.78 | 12.24 | 905.80 | 7179.91 | 278.41 | 275.70 | 554.11 |
| Yemen | 38.29 | 681.80 | 4807.31 | 185.39 | 847.93 | 1033.32 | 18.04 | 608.20 | 3946.64 | 152.76 | 378.47 | 531.23 |
| **Central Europe** | | | | | | | | | | | | |
| Albania | 11.72 | 418.16 | 2613.12 | 101.26 | 200.68 | 301.94 | 3.41 | 453.34 | 2828.38 | 110.68 | 55.19 | 165.88 |
| Bosnia and Herzegovina | 2.75 | 638.77 | 4597.21 | 178.35 | 48.18 | 226.53 | 0.99 | 636.76 | 4466.42 | 173.78 | 16.01 | 189.79 |
| Bulgaria | 2.36 | 580.52 | 4196.23 | 163.15 | 50.59 | 213.74 | 0.37 | 535.93 | 3612.72 | 141.71 | 8.16 | 149.87 |
| Croatia | 4.48 | 694.89 | 5327.11 | 204.61 | 77.47 | 282.08 | 0.59 | 585.73 | 4018.57 | 156.78 | 9.54 | 166.32 |
| Czechia | 2.02 | 471.45 | 3146.19 | 123.17 | 56.62 | 179.79 | 0.59 | 472.29 | 3053.07 | 119.70 | 14.23 | 133.94 |
| Hungary | 2.32 | 559.96 | 4067.31 | 158.20 | 60.67 | 218.87 | 0.58 | 501.38 | 3307.09 | 129.97 | 13.66 | 143.64 |
| Montenegro | 0.53 | 495.01 | 3256.97 | 128.14 | 10.93 | 139.08 | 0.34 | 537.87 | 3620.93 | 141.95 | 6.42 | 148.37 |
| Macedonia | 8.85 | 848.06 | 7017.14 | 268.59 | 149.30 | 417.89 | 2.16 | 709.20 | 5199.79 | 200.80 | 33.79 | 234.59 |
| Poland | 7.05 | 1065.27 | 9777.56 | 374.66 | 141.71 | 516.38 | 0.73 | 780.71 | 5412.72 | 211.87 | 14.49 | 226.36 |
| Romania | 4.41 | 597.20 | 4547.38 | 176.11 | 84.40 | 260.51 | 0.65 | 601.56 | 4179.67 | 163.99 | 12.02 | 176.01 |
| Serbia | 6.04 | 483.07 | 3180.46 | 123.74 | 122.25 | 245.99 | 2.18 | 451.53 | 2849.13 | 111.45 | 37.73 | 149.18 |
| Slovakia | 1.78 | 455.71 | 2996.21 | 117.02 | 42.32 | 159.34 | 0.59 | 455.76 | 2914.98 | 114.44 | 12.65 | 127.09 |
| Slovenia | 2.18 | 748.63 | 5944.55 | 229.02 | 46.43 | 275.45 | 0.42 | 645.40 | 4591.92 | 179.49 | 8.09 | 187.58 |
| **Eastern Europe** | | | | | | | | | | | | |
| Belarus | 7.18 | 685.00 | 5389.71 | 208.82 | 157.14 | 365.96 | 0.80 | 534.85 | 3417.66 | 134.38 | 17.90 | 152.27 |
| Es-nia | 4.02 | 431.52 | 2800.52 | 109.15 | 99.09 | 208.25 | 1.06 | 363.10 | 2075.20 | 81.85 | 22.03 | 103.88 |
| Latvia | 4.76 | 610.39 | 4930.20 | 190.81 | 118.54 | 309.35 | 0.86 | 508.56 | 3184.73 | 125.34 | 18.67 | 144.01 |
| Lithuania | 2.75 | 469.52 | 3288.95 | 127.55 | 69.94 | 197.50 | 0.63 | 448.71 | 2718.44 | 106.99 | 14.05 | 121.04 |
| Moldova | 3.21 | 530.18 | 3628.57 | 140.92 | 77.46 | 218.38 | 0.53 | 453.48 | 2750.93 | 108.37 | 12.02 | 120.40 |
| Russian Federation | 4.91 | 657.99 | 4811.50 | 185.34 | 127.11 | 312.45 | 0.78 | 434.05 | 2520.13 | 99.32 | 19.09 | 118.41 |
| Ukraine | 1.97 | 682.78 | 4703.49 | 184.02 | 43.60 | 227.61 | 0.41 | 552.68 | 3227.13 | 127.70 | 11.16 | 138.87 |
| **Western Europe** | | | | | | | | | | | | |
| Cyprus | 8.26 | 568.60 | 7624.90 | 296.12 | 110.58 | 406.71 | 1.93 | 546.48 | 7088.53 | 276.03 | 23.33 | 299.36 |
| Greece | 1.24 | 464.12 | 5885.14 | 227.92 | 25.22 | 253.14 | 0.26 | 451.83 | 5301.47 | 207.03 | 5.87 | 212.91 |
| Israel | 4.43 | 519.52 | 6611.94 | 257.37 | 99.63 | 357.00 | 1.11 | 439.70 | 4958.02 | 194.08 | 22.32 | 216.41 |

**Table S3 The trends of age-standardized rates of incidence, mortality, prevanlence YLLs and DALYs in 1990-2019 in “the Belt & Road” countries.**

|  | **Incidence** | | **Mortality** | | **Prevanlence** | | **YLL** | | **DALYs** | |
| --- | --- | --- | --- | --- | --- | --- | --- | --- | --- | --- |
| **Countries** | **AAPC *95%CI*** | ***P* value** | **AAPC *95%CI*** | ***P* value** | **AAPC *95%CI*** | ***P* value** | **AAPC *95%CI*** | ***P* value** | **AAPC 95%CI** | ***P value*** |
| Global | -0.47(-0.607--0.27) | 0.078 | -2.65(-2.73--2.57) | <0.001 | -1.04(-1.26--0.81) | <0.001 | -2.77(-2.84--2.71) | <0.001 | -2.05(-2.16--1.94) | <0.001 |
| High SDI | 0.18(-0.08-0.44) | <0.001 | -5.35(-5.76--4.94) | <0.001 | -0.50(-0.75--0.25) | <0.001 | -4.81(-5.20--4.43) | <0.001 | -1.10(-1.36--0.85) | <0.001 |
| High-middle SDI | -0.81(-0.56--1.06) | <0.001 | -4.16(-4.32--4.00) | <0.001 | -1.48(-1.73--1.23) | <0.001 | -4.68(-4.89--4.47) | <0.001 | -2.52(-2.76--2.27) | <0.001 |
| Middle SDI | -0.35(-0.55--0.14) | 0.00 | -3.25(-3.34--3.15) | <0.001 | -0.57(-0.78--0.34) | <0.001 | -3.23(-3.30--3.17) | <0.001 | -2.20(-2.29--2.11) | <0.001 |
| Low-middle SDI | -0.36(-0.58--0.14) | 0.00 | -2.46(-2.55--2.36) | <0.001 | -0.54(-0.80--0.27) | <0.001 | -2.74(-2.82--2.66) | <0.001 | -2.36(-2.44--2.28) | <0.001 |
| Low SDI | -0.29(-0.38--0.21) | <0.001 | -2.03(-2.16--1.90) | <0.001 | -0.55(-0.45--0.65) | <0.001 | -2.33(-2.21--2.45) | <0.001 | -1.99(-2.07--1.91) | <0.001 |
| **East Asia** | | | | | | | | | | |
| China | -0.59(-1.25-0.07) | <0.001 | -5.22(-5.45--5.00) | <0.001 | -0.97(-1.58--0.35) | 0.003 | -5.71(-5.92--5.50) | <0.001 | -2.89(-3.36--2.42) | <0.001 |
| **Central Asia** | | | | | | | | | | |
| Armenia | 0.21(0.11-0.30) | <0.001 | -6.59(-6.95--6.24) | <0.001 | 0.28(0.15-0.40) | <0.001 | -6.96(-7.30--6.63) | <0.001 | -1.46(-1.64--1.27) | <0.001 |
| Azerbaijan | -0.13(-0.27-0.02) | 0.079 | -2.67(-3.12--2.22) | <0.001 | -0.37(-0.57--0.18) | <0.001 | -3.38(-3.84--2.91) | <0.001 | -2.37(-2.75--1.99) | <0.001 |
| Georgia | 0.09(-0.06-0.24) | 0.234 | -4.22(-4.81--3.63) | <0.001 | -0.30(-0.43--0.16) | <0.001 | -4.14(-4.69--3.59) | <0.001 | -2.24(-2.53--1.95) | <0.001 |
| Kazakhstan | 0.10(-0.10-0.31) | 0.319 | -1.85(-2.39--1.32) | <0.001 | -0.03(-0.27-0.21) | 0.822 | -2.38(-2.89--1.86) | <0.001 | -1.90(-2.33--1.47) | <0.001 |
| Kyrgyzstan | -0.79(-0.93--0.65) | <0.001 | -5.66(-6.21--5.11) | <0.001 | -1.49(-1.63--1.35) | <0.001 | -6.01(-6.55--5.47) | <0.001 | -3.26(-3.50--3.02) | <0.001 |
| Mongolia | -0.54(-0.62--0.46) | <0.001 | -4.26(-4.68--3.83) | <0.001 | -0.90(-0.99--0.81) | <0.001 | -4.53(-4.95--4.11) | <0.001 | -3.43(-3.75--3.11) | <0.001 |
| Tajikistan | -0.27(-0.37--0.17) | <0.001 | -1.45(-1.61--1.28) | <0.001 | -0.69(-0.84--0.54) | <0.001 | -2.12(-2.29--1.96) | <0.001 | -1.65(-1.80--1.51) | <0.001 |
| Turkmenistan | -0.99(-1.24--0.74) | <0.001 | -8.46(-9.05--7.87) | <0.001 | -1.59(-1.95--1.24) | <0.001 | -8.36(-8.92--7.79) | <0.001 | -5.14(-5.67--4.61) | <0.001 |
| Uzbekistan | -0.95(-1.07--0.83) | <0.001 | -2.71(-3.21--2.21) | <0.001 | -1.66(-1.83--1.49) | <0.001 | -3.30(-3.84--2.74) | <0.001 | -2.67(-3.07--2.27) | <0.001 |
| **South Asia** | | | | | | | | | | |
| Bangladesh | -1.28(-1.41--1.15) | <0.001 | -4.51(-4.79--4.24) | <0.001 | -1.57(-1.76--1.38) | <0.001 | -4.90(-5.09--4.71) | <0.001 | -4.43(-4.60--4.27) | <0.001 |
| Bhutan | -1.35(-1.43--1.26) | <0.001 | -2.94(-3.01--2.88) | <0.001 | -1.56(-1.68--1.45) | <0.001 | -3.55(-3.65--3.46) | <0.001 | -3.31(-3.41--3.21) | <0.001 |
| India | 0.27(-0.25-0.80) | 0.298 | -2.70(-2.83--2.56) | <0.001 | 0.31(-0.25-0.87) | 0.262 | -2.81(-2.92--2.70) | <0.001 | -2.39(-2.48--2.30) | <0.001 |
| Nepal | -0.85(-1.04--0.67) | <0.001 | -1.86(-2.05--1.67) | <0.001 | -0.95(-1.18--0.71) | <0.001 | -2.28(-2.51--2.06) | <0.001 | -2.22(-2.44--2.01) | <0.001 |
| Pakistan | -0.61(-0.72--0.49) | <0.001 | -2.12(-2.44--1.81) | <0.001 | -0.80(-0.90--0.69) | <0.001 | -2.24(-2.55--1.93) | <0.001 | -2.06(-2.33--1.78) | <0.001 |
| **Southeast Asia** | | | | | | | | | | |
| Cambodia | 0.34(0.20-0.49) | <0.001 | -2.28(-2.34--2.23) | <0.001 | 0.31(0.17-0.46) | <0.001 | -2.81(-2.86--2.75) | <0.001 | -2.27(-2.32--2.22) | <0.001 |
| Indonesia | -0.17(-0.31--0.02) | 0.026 | -1.86(-1.99--1.73) | <0.001 | -0.32(-0.44--0.20) | <0.001 | -2.23(-2.33--2.14) | <0.001 | -1.78(-1.86--1.70) | <0.001 |
| Lao | -0.59(-0.67--0.51) | <0.001 | -3.41(-3.50--3.32) | <0.001 | -0.58(-0.68--0.48) | <0.001 | -3.99(-4.07--3.90) | <0.001 | -3.66(-3.74--3.59) | <0.001 |
| Malaysia | -0.46(-0.53--0.39) | <0.001 | -6.47(-7.16--5.77) | <0.001 | -1.14(-1.23--1.04) | <0.001 | -6.38(-7.01--5.75) | <0.001 | -4.35(-4.75--3.96) | <0.001 |
| Maldives | -1.55(-1.72--1.38) | <0.001 | -6.56(-6.88--6.25) | <0.001 | -1.88(-2.10--1.65) | <0.001 | -7.55(-7.91--7.20) | <0.001 | -5.42(-5.81--5.04) | <0.001 |
| Burma | (0.00-0.00) |  | -3.53(-3.64--3.42) | <0.001 | -0.43(-0.55--0.32) | <0.001 | -3.99(-4.14--3.84) | <0.001 | -3.74(-3.87--3.60) | <0.001 |
| Philippines | -0.70(-0.77--0.64) | <0.001 | -2.25(-2.38--2.12) | <0.001 | -0.90(-0.93--0.86) | <0.001 | -1.92(-2.07--1.78) | <0.001 | -1.64(-1.75--1.54) | <0.001 |
| Sri Lanka | 0.03(-0.02-0.09) | 0.205 | -1.71(-1.98--1.44) | <0.001 | -0.28(-0.35--0.21) | <0.001 | -2.58(-2.77--2.39) | <0.001 | -2.28(-2.44--2.13) | <0.001 |
| Thailand | -0.61(-0.79--0.44) | <0.001 | -4.67(-4.88--4.46) | <0.001 | -0.98(-1.18--0.78) | <0.001 | -4.77(-4.98--4.57) | <0.001 | -3.06(-3.23--2.88) | <0.001 |
| Viet Nam | 0.38(0.16-0.60) | 0.002 | -2.95(-2.99--2.91) | <0.001 | 0.47(0.32-0.63) | <0.001 | -3.11(-3.18--3.03) | <0.001 | -2.03(-2.11--1.94) | <0.001 |
| **High-income Asia pacific** | | | | | | | | | | |
| Brunei | -0.50(-0.59--0.42) | <0.001 | -3.61(-3.78--3.43) | <0.001 | -0.82(-0.93--0.72) | <0.001 | -4.24(-4.41--4.06) | <0.001 | -2.68(-2.81--2.56) | <0.001 |
| Singapore | -1.27(-1.44--1.09) | <0.001 | -8.74(-9.33--8.16) | <0.001 | -2.36(-2.66--2.06) | <0.001 | -9.26(-9.77--8.75) | <0.001 | -3.75(-4.16--3.34) | <0.001 |
| **North Africa and Middle East** | | | | | | | | | | |
| Afghanistan | -0.15(-0.21--0.09) | <0.001 | -2.21(-2.55--1.87) | <0.001 | -0.43(-0.48--0.38) | <0.001 | -2.45(-2.81--2.08) | <0.001 | -2.10(-2.40--1.80) | <0.001 |
| Bahrain | -0.55(-0.62--0.48) | <0.001 | -4.15(-4.45--3.85) | <0.001 | -0.93(-1.03--0.83) | <0.001 | -4.74(-5.03--4.46) | <0.001 | -2.96(-3.13--2.79) | <0.001 |
| Egypt | -0.32(-0.44--0.20) | <0.001 | -2.31(-2.41--2.20) | <0.001 | -0.34(-0.46--0.22) | <0.001 | -2.66(-2.77--2.55) | <0.001 | -2.12(-2.22--2.01) | <0.001 |
| Iran | -0.85(-0.95--0.76) | <0.001 | -4.40(-4.61--4.18) | <0.001 | -1.20(-1.33--1.08) | <0.001 | -4.53(-4.73--4.33) | <0.001 | -3.11(-3.25--2.97) | <0.001 |
| Iraq | -0.89(-0.93--0.85) | <0.001 | -3.84(-4.16--3.52) | <0.001 | -1.45(-1.50--1.40) | <0.001 | -4.17(-4.53--3.81) | <0.001 | -3.01(-3.21--2.80) | <0.001 |
| Jordan | -0.08(-0.14--0.02) | 0.015 | -5.00(-5.32--4.68) | <0.001 | -0.30(-0.37--0.23) | <0.001 | -5.30(-5.67--4.92) | <0.001 | -2.29(-2.45--2.13) | <0.001 |
| Kuwait | -0.11(-0.20--0.01) | 0.033 | -4.69(-5.43--3.95) | <0.001 | -0.33(-0.43--0.23) | <0.001 | -5.12(-5.85--4.37) | <0.001 | -1.55(-1.71--1.40) | <0.001 |
| Lebanon | -0.02(-0.07-0.03) | 0.402 | -3.61(-3.66--3.56) | <0.001 | -0.16(-0.22--0.10) | <0.001 | -3.72(-3.79--3.64) | <0.001 | -1.96(-2.06--1.85) | <0.001 |
| Oman | 0.99(0.82-1.17) | <0.001 | -3.08(-3.49--2.67) | <0.001 | 1.04(0.84-1.24) | <0.001 | -3.83(-4.13--3.53) | <0.001 | 0.36(0.12-0.61) | <0.001 |
| Palestine | -0.04(-0.19-0.10) | 0.544 | -3.08(-3.22--2.95) | <0.001 | -0.37(-0.55--0.20) | <0.001 | -3.45(-3.58--3.32) | <0.001 | -2.06(-2.20--1.92) | <0.001 |
| Qatar | -0.16(-0.24--0.08) | <0.001 | -4.50(-4.77--4.24) | <0.001 | -0.17(-0.30--0.05) | 0.008 | -5.13(-5.37--4.89) | <0.001 | -1.63(-1.81--1.45) | <0.001 |
| Saudi Arabia | 0.79(0.69-0.89) | <0.001 | -3.06(-3.17--2.95) | <0.001 | 0.78(0.66-0.89) | <0.001 | -3.14(-3.28--2.99) | <0.001 | -1.97(-2.06--1.87) | <0.001 |
| Syrian Arab Republic | 0.25(-0.05-0.55) | 0.094 | -2.38(-2.58--2.17) | <0.001 | 0.10(-0.15-0.35) | 0.431 | -2.46(-2.72--2.21) | <0.001 | -1.79(-2.04--1.54) | <0.001 |
| Turkey | -0.41(-0.61--0.21) | <0.001 | -3.57(-3.88--3.26) | <0.001 | -0.74(-1.01--0.47) | <0.001 | -4.86(-5.15--4.58) | <0.001 | -2.25(-2.53--1.98) | <0.001 |
| United Arab Emirates | 0.23(-0.13-0.59) | 0.200 | -3.69(-4.09--3.28) | <0.001 | -0.30(-0.63-0.03) | 0.075 | -3.64(-3.91--3.37) | <0.001 | -2.31(-2.54--2.07) | <0.001 |
| Yemen | -0.47(-0.54--0.40) | <0.001 | -2.99(-3.14--2.84) | <0.001 | -0.77(-0.85--0.70) | <0.001 | -3.17(-3.32--3.01) | <0.001 | -2.61(-2.75--2.47) | <0.001 |
| **Central Europe** | | | | | | | | | | |
| Albania | 0.33(0.18-0.48) | <0.001 | -5.05(-5.51--4.59) | <0.001 | 0.19(0.04-0.34) | 0.016 | -5.25(-5.73--4.77) | <0.001 | -2.53(-2.80--2.25) | <0.001 |
| Bosnia and Herzegovina | 0.02(0.00-0.04) | 0.020 | -3.82(-4.06--3.57) | <0.001 | -0.06(-0.10--0.02) | 0.008 | -4.18(-4.42--3.94) | <0.001 | -0.67(-0.74--0.60) | <0.001 |
| Bulgaria | -0.31(-0.44--0.18) | <0.001 | -7.99(-8.65--7.33) | <0.001 | -0.66(-0.78--0.53) | <0.001 | -7.85(-8.49--7.21) | <0.001 | -1.57(-1.76--1.37) | <0.001 |
| Croatia | -0.81(-0.95--0.67) | <0.001 | -7.70(-8.06--7.34) | <0.001 | -1.32(-1.50--1.14) | <0.001 | -8.02(-8.39--7.64) | <0.001 | -2.20(-2.44--1.96) | <0.001 |
| Czechia | -0.09(-0.20-0.01) | 0.082 | -3.88(-4.37--3.39) | <0.001 | -0.29(-0.46--0.11) | 0.002 | -4.37(-4.82--3.92) | <0.001 | -1.03(-1.28--0.79) | <0.001 |
| Hungary | -0.41(-0.59--0.22) | <0.001 | -5.17(-5.40--4.93) | <0.001 | -0.87(-1.02--0.72) | <0.001 | -5.60(-5.85--5.35) | <0.001 | -1.67(-1.84--1.50) | <0.001 |
| Montenegro | 0.28(0.19-0.37) | <0.001 | -1.70(-1.97--1.43) | <0.001 | 0.33(0.20-0.47) | <0.001 | -2.10(-2.46--1.73) | <0.001 | 0.17(0.05-0.28) | <0.001 |
| Macedonia | -0.95(-1.14--0.76) | <0.001 | -5.51(-5.81--5.21) | <0.001 | -1.55(-1.82--1.28) | <0.001 | -5.89(-6.21--5.56) | <0.001 | -2.53(-2.78--2.29) | <0.001 |
| Poland | -1.41(-1.56--1.25) | <0.001 | -8.38(-8.93--7.83) | <0.001 | -2.53(-2.78--2.29) | <0.001 | -8.40(-8.90--7.90) | <0.001 | -3.28(-3.58--2.98) | <0.001 |
| Romania | 0.01(-0.15-0.17) | 0.883 | -6.95(-7.23--6.66) | <0.001 | -0.40(-0.52--0.28) | <0.001 | -7.17(-7.47--6.87) | <0.001 | -1.46(-1.57--1.34) | <0.001 |
| Serbia | -0.34(-0.38--0.30) | <0.001 | -4.32(-4.74--3.89) | <0.001 | -0.53(-0.62--0.45) | <0.001 | -4.92(-5.37--4.46) | <0.001 | -2.20(-2.42--1.97) | <0.001 |
| Slovakia | -0.04(-0.10-0.02) | 0.225 | -3.98(-4.25--3.72) | <0.001 | -0.17(-0.28--0.06) | 0.004 | -4.32(-4.58--4.06) | <0.001 | -0.85(-0.96--0.74) | <0.001 |
| Slovenia | -0.61(-0.66--0.56) | <0.001 | -6.77(-7.40--6.14) | <0.001 | -1.09(-1.17--1.00) | <0.001 | -7.19(-7.81--6.56) | <0.001 | -1.62(-1.76--1.49) | <0.001 |
| **Eastern Europe** | | | | | | | | | | |
| Belarus | -1.19(-1.38--1.00) | <0.001 | -8.90(-9.52--8.28) | <0.001 | -2.01(-2.24--1.78) | <0.001 | -8.88(-9.53--8.23) | <0.001 | -3.80(-4.13--3.48) | <0.001 |
| Es-nia | -1.17(-1.48--0.86) | <0.001 | -5.21(-5.49--4.94) | <0.001 | -1.73(-2.10--1.37) | <0.001 | -5.94(-6.27--5.61) | <0.001 | -3.23(-3.57--2.88) | <0.001 |
| Latvia | -0.53(-0.66--0.39) | <0.001 | -6.70(-7.10--6.29) | <0.001 | -1.47(-1.68--1.27) | <0.001 | -7.31(-7.77--6.84) | <0.001 | -2.93(-3.24--2.62) | <0.001 |
| Lithuania | 0.02(-0.13-0.17) | 0.762 | -5.51(-5.87--5.14) | <0.001 | -0.62(-0.78--0.47) | <0.001 | -6.01(-6.40--5.62) | <0.001 | -1.83(-2.08--1.58) | <0.001 |
| Moldova | -0.69(-0.78--0.60) | <0.001 | -7.10(-7.75--6.45) | <0.001 | -1.18(-1.32--1.04) | <0.001 | -7.24(-7.88--6.60) | <0.001 | -2.56(-2.74--2.37) | <0.001 |
| Russian Federation | -1.92(-2.13--1.71) | <0.001 | -7.73(-8.27--7.18) | <0.001 | -2.74(-2.94--2.55) | <0.001 | -7.96(-8.52--7.41) | <0.001 | -4.26(-4.58--3.94) | <0.001 |
| Ukraine | -1.08(-1.28--0.89) | <0.001 | -7.38(-8.12--6.63) | <0.001 | -1.86(-2.13--1.59) | <0.001 | -6.73(-7.57--5.89) | <0.001 | -2.49(-2.82--2.16) | <0.001 |
| **Western Europe** | | | | | | | | | | |
| Cyprus | 0.01(-0.07-0.09) | 0.823 | -5.85(-6.10--5.59) | <0.001 | -0.12(-0.19--0.05) | 0.001 | -6.23(-6.51--5.94) | <0.001 | -1.15(-1.24--1.05) | <0.001 |
| Greece | 0.12(0.03-0.20) | 0.010 | -5.11(-5.58--4.64) | <0.001 | -0.19(-0.27--0.11) | <0.001 | -4.81(-5.24--4.39) | <0.001 | -0.40(-0.50--0.30) | <0.001 |
| Israel | -0.70(-0.82--0.59) | <0.001 | -5.36(-5.80--4.93) | <0.001 | -1.22(-1.42--1.02) | <0.001 | -5.80(-6.21--5.40) | <0.001 | -2.02(-2.27--1.76) | <0.001 |

**Table S4 The trends of age-standardized rates of YLDs in 1990-2019 and 2010-2019 in “the Belt & Road” countries.**

|  | **YLD 2010-2019** | | **YLD 1990-2019** | |
| --- | --- | --- | --- | --- |
| **Countries** | **AAPC *95%CI*** | ***P* value** | **AAPC 95%CI** | ***P value*** |
| Global | 0.43(-0.12-0.97) | 0.107 | -1.01(-1.23--0.78) | <0.001 |
| High SDI | 0.07(-0.24-0.39) | 0.618 | -0.47(-0.71--0.23) | <0.001 |
| Middle SDI | 1.32(0.71-1.93) | 0.001 | -0.54(-0.76--0.32) | <0.001 |
| Low SDI | 0.18(-0.24-0.60) | 0.347 | -0.53(-0.63--0.43) | <0.001 |
| **East Asia** | | | | |
| China | 4.74(2.63-6.88) | <0.001 | -0.92(-1.54--0.30) | 0.005 |
| **Central Asia** | | | | |
| Armenia | 1.24(0.73-1.76) | <0.001 | 0.30(0.18-0.42) | <0.001 |
| Azerbaijan | 1.26(0.85-1.66) | <0.001 | -0.35(-0.55--0.16) | 0.001 |
| Georgia | 0.35(-0.36-1.08) | 0.289 | -0.26(-0.40--0.13) | <0.001 |
| Kazakhstan | 1.75(1.09-2.42) | <0.001 | 0.00(-0.24-0.24) | 0.982 |
| Kyrgyzstan | -0.32(-0.74-0.11) | 0.121 | -1.44(-1.59--1.30) | <0.001 |
| Mongolia | 0.16(-0.30-0.62) | 0.450 | -0.85(-0.94--0.76) | <0.001 |
| Tajikistan | 0.88(0.34-1.42) | 0.005 | -0.66(-0.82--0.51) | <0.001 |
| Turkmenistan | 1.30(0.67-1.93) | 0.001 | -1.56(-1.91--1.21) | <0.001 |
| Uzbekistan | -0.36(-0.88-0.16) | 0.150 | -1.63(-1.80--1.46) | <0.001 |
| **South Asia** | | | | |
| Bangladesh | -0.16(-0.56-0.24) | 0.381 | -1.55(-1.74--1.36) | <0.001 |
| Bhutan | -0.90(-1.32--0.47) | 0.001 | -1.96(-2.19--1.73) | <0.001 |
| India | 1.23(0.36-2.12) | 0.012 | 0.37(-0.20-0.94) | 0.199 |
| Nepal | 1.24(0.83-1.64) | <0.001 | -0.93(-1.15--0.70) | <0.001 |
| Pakistan | -0.22(-0.66-0.23) | 0.295 | -0.78(-0.89--0.68) | <0.001 |
| **Southeast Asia** | | | | |
| Cambodia | 1.66(1.18-2.13) | <0.001 | 0.34(0.19-0.49) | <0.001 |
| Indonesia | -0.59(-1.05--0.13) | 0.018 | -0.28(-0.40--0.16) | <0.001 |
| Lao | 0.16(-0.13-0.45) | 0.243 | -0.57(-0.67--0.47) | <0.001 |
| Malaysia | -0.95(-1.59--0.30) | 0.010 | -1.06(-1.16--0.97) | <0.001 |
| Maldives | 0.27(-0.57-1.11) | 0.485 | -1.82(-2.05--1.60) | <0.001 |
| Burma | 0.55(0.23-0.87) | 0.004 | -0.42(-0.53--0.30) | <0.001 |
| Philippines | -0.56(-0.68--0.44) | <0.001 | -0.88(-0.91--0.85) | <0.001 |
| Sri Lanka | 0.04(-0.26-0.34) | 0.778 | -0.30(-0.37--0.23) | <0.001 |
| Thailand | 0.19(-0.63-1.02) | 0.608 | -0.93(-1.13--0.72) | <0.001 |
| Viet Nam | 1.19(1.16-1.23) | <0.001 | 0.48(0.32-0.65) | <0.001 |
| **High-income Asia pacific** | | | | |
| Brunei | -0.43(-0.51--0.35) | <0.001 | -0.79(-0.89--0.69) | <0.001 |
| Singapore | -0.40(-0.68--0.12) | 0.011 | -2.29(-2.59--1.99) | <0.001 |
| **North Africa and Middle East** | | | | |
| Afghanistan | 0.06(-0.21-0.32) | 0.635 | -0.41(-0.46--0.35) | <0.001 |
| Bahrain | -0.29(-0.89-0.30) | 0.288 | -0.90(-0.99--0.80) | <0.001 |
| Egypt | 0.74(0.38-1.10) | 0.001 | -0.34(-0.46--0.22) | <0.001 |
| Iran | -0.37(-0.58--0.16) | 0.004 | -1.17(-1.29--1.04) | <0.001 |
| Iraq | -1.02(-1.39--0.65) | <0.001 | -1.41(-1.46--1.36) | <0.001 |
| Jordan | 0.22(-0.33-0.77) | 0.380 | -0.27(-0.34--0.20) | <0.001 |
| Kuwait | 0.05(-0.42-0.52) | 0.818 | -0.31(-0.41--0.21) | <0.001 |
| Lebanon | 0.34(0.07-0.60) | 0.019 | -0.15(-0.21--0.09) | <0.001 |
| Oman | 2.03(1.29-2.77) | <0.001 | 1.06(0.86-1.25) | <0.001 |
| Palestine | 0.91(0.56-1.27) | <0.001 | -0.35(-0.53--0.18) | <0.001 |
| Qatar | 1.23(0.99-1.46) | <0.001 | -0.18(-0.31--0.05) | 0.008 |
| Saudi Arabia | 0.89(0.24-1.53) | 0.013 | 0.80(0.68-0.91) | <0.001 |
| Syrian Arab Republic | -0.53(-0.83--0.23) | 0.004 | 0.11(-0.15-0.37) | 0.410 |
| Turkey | 2.34(1.67-3.02) | <0.001 | -0.76(-1.03--0.48) | <0.001 |
| United Arab Emirates | -3.27(-4.11--2.43) | <0.001 | -0.28(-0.61-0.06) | 0.105 |
| Yemen | -0.15(-0.26--0.04) | 0.015 | -0.76(-0.83--0.69) | <0.001 |
| **Central Europe** | | | | |
| Albania | 0.76(-0.40-1.94) | 0.171 | 0.24(0.09-0.40) | 0.003 |
| Bosnia and Herzegovina | 0.07(-0.04-0.18) | 0.200 | -0.04(-0.08-0.00) | 0.048 |
| Bulgaria | 0.16(-0.26-0.57) | 0.414 | -0.79(-0.89--0.69) | <0.001 |
| Croatia | 0.03(-0.42-0.48) | 0.883 | -1.26(-1.44--1.08) | <0.001 |
| Czechia | 0.96(0.53-1.40) | 0.001 | -0.28(-0.45--0.11) | 0.002 |
| Hungary | -0.36(-0.71-0.00) | 0.048 | -0.83(-0.98--0.67) | <0.001 |
| Montenegro | 1.51(0.84-2.19) | 0.001 | 0.33(0.20-0.46) | <0.001 |
| Macedonia | -0.29(-0.41--0.18) | <0.001 | -1.50(-1.76--1.23) | <0.001 |
| Poland | -1.02(-1.41--0.62) | <0.001 | -2.46(-2.70--2.21) | <0.001 |
| Romania | 0.03(-0.31-0.36) | 0.863 | -0.35(-0.47--0.22) | <0.001 |
| Serbia | -0.03(-0.32-0.26) | 0.814 | -0.50(-0.59--0.42) | <0.001 |
| Slovakia | 0.35(0.00-0.71) | 0.051 | -0.15(-0.26--0.03) | 0.012 |
| Slovenia | -0.12(-0.41-0.17) | 0.377 | -1.04(-1.12--0.95) | <0.001 |
| **Eastern Europe** | | | | |
| Belarus | 0.25(-0.33-0.84) | 0.342 | -1.96(-2.19--1.73) | <0.001 |
| Es-nia | 2.51(2.05-2.96) | <0.001 | -1.69(-2.06--1.33) | <0.001 |
| Latvia | -0.21(-0.51-0.10) | 0.155 | -1.41(-1.61--1.21) | <0.001 |
| Lithuania | 0.41(0.06-0.77) | 0.027 | -0.55(-0.71--0.40) | <0.001 |
| Moldova | 0.25(-0.13-0.62) | 0.167 | -1.12(-1.26--0.99) | <0.001 |
| Russian Federation | -0.77(-1.41--0.13) | 0.024 | -2.66(-2.85--2.46) | <0.001 |
| Ukraine | 0.81(-0.15-1.78) | 0.086 | -1.81(-2.07--1.55) | <0.001 |
| **Western Europe** | | | | |
| Cyprus | 0.16(0.03-0.30) | 0.023 | -0.11(-0.18--0.04) | 0.003 |
| Greece | -0.65(-0.82--0.49) | <0.001 | -0.16(-0.24--0.07) | <0.001 |
| Israel | -0.01(-0.36-0.35) | 0.964 | -1.20(-1.39--1.00) | <0.001 |

**Table S5 The trends of age-standardized rates of YLDs in genders in 1990-2019 in “the Belt & Road” countries.**

|  | **male** | | **Female** | |
| --- | --- | --- | --- | --- |
| **Countries** | **AAPC *95%CI*** | ***P* value** | **AAPC *95%CI*** | ***P* value** |
| Global | -0.94(-1.17--0.70) | <0.001 | -1.09(-1.30--0.88) | <0.001 |
| High SDI | -0.73(-0.95--0.51) | <0.001 | -0.25(-0.52-0.0-) | 0.076 |
| High-middle SDI | -1.45(-1.71--1.18) | <0.001 | -1.43(-1.67--1.19) | <0.001 |
| Middle SDI | -0.33(-0.55--0.11) | 0.004 | -0.78(-1.01--0.55) | <0.001 |
| Low-middle SDI | -0.27(-0.58-0.04) | <0.001 | -0.79(-1.02--0.55) | <0.001 |
| Low SDI | -0.32(-0.46--0.18) | <0.001 | -0.73(-0.80--0.65) | <0.001 |
| **East Asia** | | | | |
| China | -0.70(-1.28--0.11) | 0.021 | -1.25(-1.92--0.56) | 0.001 |
| **Central Asia** | | | | |
| Armenia | 0.26(0.15-0.37) | <0.001 | 0.32(0.19-0.45) | <0.001 |
| Azerbaijan | -0.37(-0.59--0.15) | 0.002 | -0.35(-0.53--0.17) | <0.001 |
| Georgia | -0.81(-0.92--0.70) | <0.001 | 0.34(0.11-0.57) | 0.005 |
| Kazakhstan | 0.32(0.03-0.62) | 0.032 | -0.19(-0.39-0.01) | 0.065 |
| Kyrgyzstan | -1.62(-1.73--1.51) | <0.001 | -1.27(-1.47--1.07) | <0.001 |
| Mongolia | -0.44(-0.53--0.36) | <0.001 | -1.19(-1.29--1.09) | <0.001 |
| Tajikistan | -0.64(-0.87--0.41) | <0.001 | -0.68(-0.77--0.58) | <0.001 |
| Turkmenistan | -1.33(-1.64--1.02) | <0.001 | -1.77(-2.16--1.38) | <0.001 |
| Uzbekistan | -1.44(-1.62--1.27) | <0.001 | -1.77(-1.94--1.59) | <0.001 |
| **South Asia** | | | | |
| Bangladesh | -1.47(-1.73--1.21) | <0.001 | -1.70(-1.84--1.55) | <0.001 |
| Bhutan | -1.17(-1.24--1.11) | <0.001 | -1.78(-1.93--1.63) | <0.001 |
| India | 0.86(0.20-1.52) | 0.013 | -0.26(-0.73-0.22) | 0.281 |
| Nepal | -1.49(-1.81--1.18) | <0.001 | -0.66(-0.84--0.47) | <0.001 |
| Pakistan | -1.31(-1.45--1.16) | <0.001 | -0.48(-0.56--0.40) | <0.001 |
| **Southeast Asia** | | | | |
| Cambodia | 0.54(0.40-0.69) | <0.001 | 0.15(-0.01-0.30) | 0.065 |
| Indonesia | -0.05(-0.19-0.08) | 0.421 | -0.61(-0.71--0.51) | <0.001 |
| Lao | -0.61(-0.72--0.51) | <0.001 | -0.52(-0.62--0.43) | <0.001 |
| Malaysia | -1.18(-1.31--1.04) | <0.001 | -0.95(-1.03--0.86) | <0.001 |
| Maldives | -1.09(-1.34--0.84) | <0.001 | -2.57(-2.75--2.39) | <0.001 |
| Burma | -0.40(-0.57--0.23) | <0.001 | -0.43(-0.49--0.36) | <0.001 |
| Philippines | -0.90(-0.93--0.86) | <0.001 | -0.85(-0.88--0.82) | <0.001 |
| Sri Lanka | -0.13(-0.18--0.07) | <0.001 | -0.44(-0.52--0.35) | <0.001 |
| Thailand | -0.69(-0.89--0.50) | <0.001 | -1.15(-1.35--0.94) | <0.001 |
| Viet Nam | 0.67(0.48-0.87) | <0.001 | 0.22(0.08-0.37) | 0.004 |
| **High-income Asia pacific** | | | | |
| Brunei | -0.93(-1.05--0.80) | <0.001 | -0.63(-0.72--0.55) | <0.001 |
| Singapore | -2.62(-2.93--2.30) | <0.001 | -1.95(-2.22--1.68) | <0.001 |
| **North Africa and Middle East** | | | | |
| Afghanistan | -0.04(-0.15-0.08) | 0.531 | -0.55(-0.58--0.51) | <0.001 |
| Bahrain | -0.60(-0.69--0.50) | <0.001 | -1.15(-1.30--1.01) | <0.001 |
| Egypt | -0.23(-0.34--0.12) | <0.001 | -0.41(-0.55--0.27) | <0.001 |
| Iran | -1.12(-1.22--1.01) | <0.001 | -1.23(-1.37--1.08) | <0.001 |
| Iraq | -1.87(-1.94--1.80) | <0.001 | -0.92(-0.96--0.87) | <0.001 |
| Jordan | -0.31(-0.40--0.22) | <0.001 | -0.22(-0.29--0.14) | <0.001 |
| Kuwait | -0.43(-0.53--0.34) | <0.001 | -0.22(-0.34--0.10) | 0.001 |
| Lebanon | -0.09(-0.13--0.05) | <0.001 | -0.21(-0.29--0.13) | <0.001 |
| Oman | 1.04(0.85-1.24) | <0.001 | 1.09(0.89-1.30) | <0.001 |
| Palestine | -0.39(-0.54--0.25) | <0.001 | -0.29(-0.49--0.09) | 0.006 |
| Qatar | 0.03(-0.11-0.16) | 0.706 | -0.14(-0.24--0.04) | 0.009 |
| Saudi Arabia | 0.94(0.84-1.04) | <0.001 | 0.57(0.43-0.71) | <0.001 |
| Syrian Arab Republic | 0.19(-0.11-0.49) | 0.202 | 0.00(-0.23-0.22) | 0.971 |
| Turkey | -0.65(-0.90--0.39) | <0.001 | -0.85(-1.15--0.54) | <0.001 |
| United Arab Emirates | -0.65(-0.86--0.44) | <0.001 | 0.32(-0.12-0.77) | 0.147 |
| Yemen | -0.57(-0.62--0.53) | <0.001 | -0.93(-1.02--0.84) | <0.001 |
| **Central Europe** | | | | |
| Albania | -0.17(-0.29--0.05) | 0.009 | 0.64(0.40-0.88) | <0.001 |
| Bosnia and Herzegovina | -0.10(-0.13--0.07) | <0.001 | -0.03(-0.10-0.03) | 0.295 |
| Bulgaria | -0.59(-0.71--0.46) | <0.001 | -0.64(-0.77--0.51) | <0.001 |
| Croatia | -1.41(-1.58--1.24) | <0.001 | -1.13(-1.35--0.92) | <0.001 |
| Czechia | -0.12(-0.33-0.10) | 0.289 | -0.40(-0.54--0.26) | <0.001 |
| Hungary | -1.11(-1.31--0.92) | <0.001 | -0.60(-0.72--0.48) | <0.001 |
| Montenegro | 0.42(0.29-0.55) | <0.001 | 0.25(0.11-0.38) | 0.001 |
| Macedonia | -1.55(-1.85--1.25) | <0.001 | -1.43(-1.66--1.19) | <0.001 |
| Poland | -2.96(-3.30--2.62) | <0.001 | -2.11(-2.30--1.93) | <0.001 |
| Romania | 0.13(-0.07-0.32) | 0.190 | -0.64(-0.73--0.55) | <0.001 |
| Serbia | -0.64(-0.70--0.58) | <0.001 | -0.43(-0.57--0.28) | <0.001 |
| Slovakia | -0.19(-0.26--0.12) | <0.001 | -0.11(-0.28-0.06) | 0.199 |
| Slovenia | -1.44(-1.58--1.30) | <0.001 | -0.76(-0.82--0.70) | <0.001 |
| **Eastern Europe** | | | | |
| Belarus | -1.88(-2.08--1.68) | <0.001 | -2.05(-2.32--1.78) | <0.001 |
| Es-nia | -1.92(-2.27--1.56) | <0.001 | -1.56(-1.94--1.18) | <0.001 |
| Latvia | -1.72(-1.90--1.54) | <0.001 | -1.14(-1.36--0.91) | <0.001 |
| Lithuania | -0.37(-0.51--0.22) | <0.001 | -0.73(-0.90--0.56) | <0.001 |
| Moldova | -1.30(-1.42--1.17) | <0.001 | -0.96(-1.10--0.82) | <0.001 |
| Russian Federation | -2.71(-2.95--2.47) | <0.001 | -2.61(-2.79--2.44) | <0.001 |
| Ukraine | -1.87(-2.16--1.59) | <0.001 | -1.69(-1.95--1.43) | <0.001 |
| **Western Europe** | | | | |
| Cyprus | -0.16(-0.23--0.09) | <0.001 | -0.09(-0.17--0.01) | 0.029 |
| Greece | -0.23(-0.31--0.15) | <0.001 | -0.11(-0.20--0.02) | 0.024 |
| Israel | -1.39(-1.55--1.24) | <0.001 | -1.00(-1.24--0.75) | <0.001 |

**Table S6 The trends of age-standardized YLDs rate stratified by age from 1990 to 2019 in “the Belt & Road” countries**

|  | **<5 yrs** | | **5-14 yrs** | | **15-49yrs** | | **50-74 yrs** | | **≥75 yrs** | |
| --- | --- | --- | --- | --- | --- | --- | --- | --- | --- | --- |
| **Countries** | **AAPC *95%CI*** | ***P* value** | **AAPC *95%CI*** | ***P* value** | **AAPC *95%CI*** | ***P* value** | **AAPC *95%CI*** | ***P* value** | **AAPC *95%CI*** | ***P* value** |
| Global | -0.29(-0.51--0.07) | 0.013 | 0.06(-0.21-0.33) | 0.674 | -0.87(-1.04--0.70) | <0.001 | -2.11(-2.37-1.85) | <0.001 | -2.32(-2.56--2.07) | <0.001 |
| High SDI | 0.72(0.55-0.89) | <0.001 | 0.56(0.34-0.78) | <0.001 | -0.34(-0.50--0.19) | <0.001 | -1.90(-2.32--1.48) | <0.001 | -2.56(-2.94--2.19) | <0.001 |
| High-middle SDI | -0.01(-0.31-0.29) | 0.953 | 0.09(-0.23-0.42) | 0.562 | -1.39(-1.62--1.16) | <0.001 | -3.42(-3.67--3.16) | <0.001 | -2.86(-3.04--2.68) | <0.001 |
| Middle SDI | -0.24(-0.55-0.07) | 0.118 | 0.08(-0.26-0.42) | 0.633 | -0.57(-0.77--0.37) | <0.001 | -1.44(-1.63--1.26) | <0.001 | -1.43(-1.52--1.35) | <0.001 |
| Low-middle SDI | -0.94(-1.24--0.64) | <0.001 | 0.08(-0.30-0.46) | 0.660 | -0.51(-0.75--0.26) | <0.001 | -0.82(-1.09--0.56) | <0.001 | -0.34(-0.54--0.14) | 0.002 |
| Low SDI | -0.61(-0.70--0.51) | <0.001 | -0.09(-0.21-0.04) | 0.183 | -0.76(-0.85--0.68) | <0.001 | -0.64(-0.77--0.50) | <0.001 | -0.29(-0.45--0.13) | 0.001 |
| **East Asia** | | | | | | | | | | |
| China | 0.94(-0.14-2.03) | 0.085 | -0.06(-1.00-0.89) | 0.895 | -1.72(-2.21--1.23) | <0.001 | -2.64(-3.00--2.29) | <0.001 | -2.42(-2.61--2.22) | <0.001 |
| **Central Asia** | | | | | | | | | | |
| Armenia | 0.31(0.20-0.42) | <0.001 | 0.37(0.28-0.45) | <0.001 | 0.72(0.63-0.81) | <0.001 | -0.44(-0.68--0.20) | 0.001 | -1.71(-1.94--1.48) | <0.001 |
| Azerbaijan | 0.27(0.12-0.41) | 0.001 | 0.27(0.14-0.39) | <0.001 | -0.18(-0.36-0.00) | 0.047 | -1.98(-2.26--1.70) | <0.001 | -0.62(-0.79--0.45) | <0.001 |
| Georgia | 0.18(0.05-0.32) | 0.009 | 1.25(0.80-1.70) | <0.001 | -0.41(-0.60--0.23) | <0.001 | -2.51(-3.14--1.88) | <0.001 | -2.54(-3.10--1.99) | <0.001 |
| Kazakhstan | 0.45(0.27-0.63) | <0.001 | 0.63(0.42-0.84) | <0.001 | 0.35(0.11-0.58) | 0.005 | -1.32(-1.59--1.05) | <0.001 | -1.12(-1.39--0.86) | <0.001 |
| Kyrgyzstan | 0.38(0.06-0.69) | 0.020 | 0.30(-0.01-0.61) | 0.056 | -1.42(-1.56--1.27) | <0.001 | -3.18(-3.37--2.98) | <0.001 | -2.56(-2.71--2.42) | <0.001 |
| Mongolia | 0.26(0.11-0.41) | 0.002 | 0.33(0.21-0.45) | <0.001 | 0.37(0.24-0.50) | <0.001 | -3.04(-3.23--2.85) | <0.001 | -3.14(-3.31--2.96) | <0.001 |
| Tajikistan | 0.28(0.16-0.39) | <0.001 | 0.25(0.14-0.37) | <0.001 | -0.10(-0.29-0.09) | 0.298 | -2.84(-3.15--2.52) | <0.001 | -0.80(-0.98--0.61) | <0.001 |
| Turkmenistan | -0.08(-0.23-0.07) | 0.304 | -0.27(-0.41--0.13) | <0.001 | -1.67(-2.00--1.34) | <0.001 | -3.14(-3.78--2.51) | <0.001 | -3.12(-3.73--2.51) | <0.001 |
| Uzbekistan | 0.30(0.19-0.41) | <0.001 | 0.02(-0.08-0.12) | 0.709 | -1.46(-1.68--1.24) | <0.001 | -3.07(-3.38--2.76) | <0.001 | -1.76(-1.92--1.60) | <0.001 |
| **South Asia** | | | | | | | | | | |
| Bangladesh | -1.05(-1.26--0.84) | <0.001 | -1.12(-1.21--1.04) | <0.001 | -1.31(-1.53--1.09) | <0.001 | -1.80(-2.05--1.54) | <0.001 | -1.09(-1.20--0.97) | <0.001 |
| Bhutan | -3.65(-4.09--3.22) | <0.001 | -1.50(-1.64--1.36) | <0.001 | -1.27(-1.41--1.12) | <0.001 | -1.28(-1.41--1.15) | <0.001 | -1.14(-1.27--1.02) | <0.001 |
| India | 0.07(-0.83-0.99) | 0.872 | 2.58(1.21-3.97) | 0.001 | 0.35(-0.26-0.96) | 0.251 | -0.11(-0.58-0.36) | 0.641 | 0.08(-0.29-0.44) | 0.661 |
| Nepal | -1.79(-2.17--1.41) | <0.001 | -0.47(-0.72--0.21) | 0.001 | -0.81(-1.00--0.62) | <0.001 | -0.97(-1.32--0.62) | <0.001 | -0.73(-1.12--0.34) | 0.001 |
| Pakistan | -0.90(-1.15--0.65) | <0.001 | -0.14(-0.39-0.12) | 0.276 | -0.50(-0.59--0.42) | <0.001 | -1.24(-1.36--1.12) | <0.001 | -1.18(-1.28--1.09) | <0.001 |
| **Southeast Asia** | | | | | | | | | | |
| Cambodia | 0.64(0.39-0.89) | <0.001 | 0.58(0.38-0.79) | <0.001 | 0.29(0.18-0.41) | <0.001 | -0.15(-0.24--0.05) | 0.006 | 0.38(0.30-0.47) | <0.001 |
| Indonesia | -1.18(-1.50--0.85) | <0.001 | 0.33(0.03-0.63) | 0.034 | 0.06(-0.03-0.16) | 0.198 | -0.84(-1.00--0.68) | <0.001 | -1.24(-1.41--1.08) | <0.001 |
| Lao | -1.46(-1.61--1.32) | <0.001 | -0.83(-0.95--0.72) | <0.001 | -0.30(-0.40--0.21) | <0.001 | -0.44(-0.51--0.37) | <0.001 | -0.09(-0.16--0.02) | 0.010 |
| Malaysia | 1.64(1.33-1.95) | <0.001 | 0.89(0.66-1.11) | <0.001 | -1.08(-1.22--0.93) | <0.001 | -3.37(-3.66--3.08) | <0.001 | -3.59(-4.06--3.12) | <0.001 |
| Maldives | -2.30(-2.55--2.04) | <0.001 | -1.49(-1.69--1.29) | <0.001 | -1.16(-1.38--0.94) | <0.001 | -3.36(-3.65--3.08) | <0.001 | -2.76(-2.97--2.55) | <0.001 |
| Burma | -0.64(-0.74--0.53) | <0.001 | 0.06(-0.07-0.20) | 0.344 | -0.45(-0.57--0.32) | <0.001 | -0.80(-0.92--0.68) | <0.001 | -0.15(-0.26--0.03) | 0.019 |
| Philippines | -1.41(-1.48--1.34) | <0.001 | -0.80(-1.00--0.61) | <0.001 | -0.68(-0.71--0.65) | <0.001 | -0.83(-1.03--0.63) | <0.001 | -1.26(-1.32--1.20) | <0.001 |
| Sri Lanka | 1.02(0.86-1.17) | <0.001 | 0.48(0.13-0.84) | 0.009 | -0.92(-1.13--0.72) | <0.001 | -0.90(-1.11--0.68) | <0.001 | 1.06(0.87-1.26) | <0.001 |
| Thailand | 0.84(0.6-1.08) | <0.001 | -0.44(-0.80--0.09) | 0.017 | -0.19(-0.48-0.10) | 0.184 | -2.65(-2.83--2.48) | <0.001 | -1.96(-2.08--1.84) | <0.001 |
| Viet Nam | 0.64(0.51-0.77) | <0.001 | 0.26(-0.37-0.88) | 0.406 | 0.52(0.43-0.61) | <0.001 | 0.17(0.02-0.33) | 0.032 | 0.87(0.81-0.93) | <0.001 |
| **High-income Asia pacific** | | | | | | | | | | |
| Brunei | -0.01(-0.05-0.02) | 0.514 | -0.11(-0.17--0.05) | 0.001 | -0.46(-0.57--0.35) | <0.001 | -1.98(-2.19--1.77) | <0.001 | -1.04(-1.34--0.74) | <0.001 |
| Singapore | -0.34(-0.53--0.16) | 0.001 | -0.19(-0.31--0.08) | 0.002 | -1.98(-2.27--1.70) | <0.001 | -4.78(-5.38--4.17) | <0.001 | -4.39(-4.65--4.13) | <0.001 |
| **North Africa and Middle East** | | | | | | | | | | |
| Afghanistan | 0.74(0.58-0.89) | <0.001 | 0.20(0.09-0.30) | 0.001 | -0.50(-0.56--0.43) | <0.001 | -0.78(-0.84--0.72) | <0.001 | -0.41(-0.52--0.30) | <0.001 |
| Bahrain | 0.61(0.53-0.69) | <0.001 | -0.15(-0.21--0.08) | <0.001 | -0.70(-0.87--0.54) | <0.001 | -3.12(-3.31--2.93) | <0.001 | -1.44(-1.59--1.29) | <0.001 |
| Egypt | -0.81(-1.06--0.55) | <0.001 | -0.46(-0.69--0.22) | <0.001 | -0.18(-0.30--0.06) | 0.004 | -0.27(-0.32--0.22) | <0.001 | -0.24(-0.31--0.17) | <0.001 |
| Iran | -0.09(-0.22-0.03) | 0.147 | -0.28(-0.42--0.14) | <0.001 | -1.08(-1.19--0.97) | <0.001 | -2.68(-2.98--2.38) | <0.001 | -1.92(-2.26--1.57) | <0.001 |
| Iraq | 0.39(0.30-0.49) | <0.001 | -0.49(-0.58--0.39) | <0.001 | -1.42(-1.47--1.36) | <0.001 | -2.80(-2.88--2.73) | <0.001 | -2.33(-2.40--2.25) | <0.001 |
| Jordan | 0.85(0.75-0.95) | <0.001 | 0.34(0.25-0.42) | <0.001 | -0.24(-0.31--0.17) | <0.001 | -1.32(-1.44--1.19) | <0.001 | -1.22(-1.34--1.11) | <0.001 |
| Kuwait | 0.66(0.54-0.79) | <0.001 | 0.18(-0.07-0.43) | 0.156 | -0.23(-0.33--0.13) | <0.001 | -1.44(-1.58--1.29) | <0.001 | -1.40(-1.52--1.27) | <0.001 |
| Lebanon | 0.79(0.68-0.89) | <0.001 | 0.15(0.03-0.27) | 0.015 | -0.34(-0.41--0.26) | <0.001 | -0.55(-0.62--0.48) | <0.001 | -0.20(-0.24--0.16) | <0.001 |
| Oman | 1.60(1.33-1.86) | <0.001 | 1.57(1.25-1.89) | <0.001 | 0.57(0.46-0.68) | <0.001 | 0.93(0.58-1.29) | <0.001 | 0.70(0.14-1.26) | 0.017 |
| Palestine | 1.02(0.82-1.22) | <0.001 | 0.64(0.42-0.87) | <0.001 | -0.38(-0.54--0.21) | <0.001 | -1.93(-2.05--1.82) | <0.001 | -0.94(-1.08--0.80) | <0.001 |
| Qatar | 0.25(0.19-0.32) | <0.001 | -0.38(-0.46--0.30) | <0.001 | -0.45(-0.81--0.08) | 0.019 | 0.28(0.04-0.51) | 0.024 | -0.06(-0.47-0.35) | 0.766 |
| Saudi Arabia | 1.26(1.11-1.41) | <0.001 | 1.39(1.28-1.51) | <0.001 | 0.46(0.20-0.72) | 0.001 | -0.23(-0.31--0.15) | <0.001 | -0.03(-0.12-0.07) | 0.573 |
| Syrian Arab Republic | 1.14(0.73-1.54) | <0.001 | 0.76(0.05-1.48) | 0.036 | 0.04(-0.14-0.23) | 0.644 | -0.99(-1.07--0.91) | <0.001 | -0.42(-0.47--0.37) | <0.001 |
| Turkey | -0.79(-0.98--0.60) | <0.001 | -0.42(-0.74--0.10) | 0.012 | -0.91(-1.39--0.43) | 0.001 | -1.37(-1.59--1.15) | <0.001 | 0.91(0.67-1.15) | <0.001 |
| United Arab Emirates | 2.31(1.38-3.25) | <0.001 | 1.12(0.37-1.87) | 0.005 | -0.54(-0.72--0.36) | <0.001 | -1.53(-1.69--1.37) | <0.001 | -0.68(-0.94--0.41) | <0.001 |
| Yemen | -0.11(-0.30-0.08) | 0.254 | -0.27(-0.38--0.15) | <0.001 | -0.70(-0.77--0.64) | <0.001 | -1.30(-1.37--1.23) | <0.001 | -1.14(-1.23--1.04) | <0.001 |
| **Central Europe** | | | | | | | | | | |
| Albania | 0.46(0.29-0.64) | <0.001 | 1.45(1.06-1.84) | <0.001 | 0.77(0.62-0.91) | <0.001 | -1.07(-1.39--0.75) | <0.001 | -1.72(-1.94--1.49) | <0.001 |
| Bosnia and Herzegovina | 0.16(0.12-0.20) | <0.001 | 0.24(0.22-0.27) | <0.001 | 0.25(0.16-0.35) | <0.001 | -0.66(-0.83--0.50) | <0.001 | -0.48(-0.59--0.36) | <0.001 |
| Bulgaria | 0.26(-0.10-0.62) | 0.147 | 0.45(0.07-0.83) | 0.020 | -0.39(-0.47--0.31) | <0.001 | -1.92(-2.09--1.75) | <0.001 | -2.69(-2.88--2.49) | <0.001 |
| Croatia | 0.05(-0.11-0.22) | 0.523 | -0.03(-0.28-0.22) | 0.787 | -0.46(-0.61--0.31) | <0.001 | -2.93(-3.17--2.68) | <0.001 | -3.59(-3.79--3.38) | <0.001 |
| Czechia | 0.03(-0.08-0.14) | 0.618 | 0.20(0.16-0.24) | <0.001 | -0.34(-0.52--0.16) | 0.001 | -0.97(-1.35--0.58) | <0.001 | -0.20(-0.55-0.16) | 0.261 |
| Hungary | 0.17(-0.17-0.52) | 0.314 | 0.33(-0.20-0.87) | 0.210 | -0.80(-0.90--0.71) | <0.001 | -2.22(-2.36--2.08) | <0.001 | -1.81(-1.95--1.67) | <0.001 |
| Montenegro | 0.10(0.05-0.15) | <0.001 | 0.18(0.15-0.20) | <0.001 | 0.28(0.19-0.38) | <0.001 | 0.66(0.29-1.03) | 0.001 | 0.61(0.01-1.20) | 0.045 |
| Macedonia | -0.04(-0.08-0.01) | 0.089 | 0.08(-0.05-0.21) | 0.229 | -0.50(-0.61--0.39) | <0.001 | -3.17(-3.68--2.66) | <0.001 | -2.63(-3.15--2.11) | <0.001 |
| Poland | 0.04(-0.24-0.31) | 0.797 | 0.59(0.45-0.73) | <0.001 | -2.52(-2.79--2.25) | <0.001 | -4.28(-4.60--3.96) | <0.001 | -4.36(-4.64--4.09) | <0.001 |
| Romania | 0.33(0.25-0.41) | <0.001 | 1.20(0.74-1.67) | <0.001 | -0.04(-0.14-0.07) | 0.474 | -1.90(-2.05--1.75) | <0.001 | -2.08(-2.19--1.96) | <0.001 |
| Serbia | 0.1(0.00-0.2) | 0.060 | -0.05(-0.22-0.11) | 0.511 | -0.12(-0.34-0.09) | 0.241 | -1.61(-1.77--1.44) | <0.001 | -1.64(-1.80--1.48) | <0.001 |
| Slovakia | 0.06(0.01-0.12) | 0.032 | 0.26(0.23-0.30) | <0.001 | 0.01(-0.21-0.23) | 0.920 | -0.98(-1.13--0.83) | <0.001 | -0.96(-1.11--0.81) | <0.001 |
| Slovenia | 0.07(0.01-0.13) | 0.021 | 0.21(0.19-0.23) | <0.001 | -0.56(-0.62--0.49) | <0.001 | -2.68(-2.88--2.47) | <0.001 | -2.72(-2.82--2.61) | <0.001 |
| **Eastern Europe** | | | | | | | | | | |
| Belarus | -0.05(-0.15-0.05) | 0.339 | -0.51(-0.85--0.17) | 0.005 | -1.97(-2.19--1.75) | <0.001 | -3.55(-3.76--3.34) | <0.001 | -3.65(-3.79--3.51) | <0.001 |
| Es-nia | 0.00(-0.09-0.09) | 0.973 | -0.69(-1.14--0.25) | 0.003 | -2.18(-2.83--1.52) | <0.001 | -3.10(-3.38--2.83) | <0.001 | -1.22(-1.43--1.01) | <0.001 |
| Latvia | 0.72(0.54-0.90) | <0.001 | 1.04(0.82-1.25) | <0.001 | -2.04(-2.28--1.80) | <0.001 | -3.23(-3.45--3.00) | <0.001 | -2.15(-2.25--2.04) | <0.001 |
| Lithuania | 1.29(1.04-1.55) | <0.001 | 1.63(1.27-1.99) | <0.001 | -0.70(-0.83--0.57) | <0.001 | -2.87(-3.15--2.59) | <0.001 | -2.28(-2.44--2.12) | <0.001 |
| Moldova | 0.07(0.01-0.13) | 0.029 | 0.04(-0.01-0.10) | 0.116 | -0.79(-0.91--0.67) | <0.001 | -3.22(-3.53--2.91) | <0.001 | -3.95(-4.29--3.61) | <0.001 |
| Russian Federation | -0.76(-1.12--0.39) | <0.001 | -0.77(-1.06--0.48) | <0.001 | -2.29(-2.41--2.17) | <0.001 | -5.62(-5.92--5.32) | <0.001 | -5.05(-5.29--4.80) | <0.001 |
| Ukraine | 0.20(0.02-0.39) | 0.032 | -0.23(-0.36--0.09) | 0.002 | -2.43(-2.73--2.13) | <0.001 | -3.82(-4.31--3.32) | <0.001 | -5.10(-5.64--4.57) | <0.001 |
| **Western Europe** | | | | | | | | | | |
| Cyprus | 0.48(0.19-0.76) | 0.002 | 0.56(0.22-0.90) | 0.002 | -0.08(-0.12--0.04) | <0.001 | -0.59(-0.69--0.50) | <0.001 | -0.77(-0.96--0.57) | <0.001 |
| Greece | 0.70(0.49-0.92) | <0.001 | 1.73(1.44-2.01) | <0.001 | 0.06(-0.03-0.15) | 0.160 | -1.41(-1.52--1.29) | <0.001 | -2.72(-2.83--2.61) | <0.001 |
| Israel | -0.29(-0.35--0.22) | <0.001 | -0.17(-0.26--0.07) | 0.001 | -0.87(-1.12--0.63) | <0.001 | -2.51(-2.75--2.27) | <0.001 | -2.72(-2.91--2.53) | <0.001 |

**Table S7 The trends of age-standardized YLDs rate Contributed to risk factors from 1990 to 2019 in “the Belt & Road” countries.**

|  | **High body-mass index** | | **Occupational risks** | | **Tobacco** | |
| --- | --- | --- | --- | --- | --- | --- |
| **Countries** | **AAPC *95%CI*** | ***P* value** | **AAPC *95%CI*** | ***P* value** | **AAPC *95%CI*** | ***P* value** |
| Global | -0.20(-0.41-0.01) | 0.065 | -1.36(-1.55--1.18) | <0.001 | -2.69(-2.90--2.49) | <0.001 |
| High SDI | 0.56(0.32-0.81) | <0.001 | -0.88(-1.1--0.66) | <0.001 | -2.21(-2.49--1.93) | <0.001 |
| Middle SDI | 1.27(1.10-1.43) | <0.001 | -1.08(-1.25--0.91) | <0.001 | -1.73(-1.89--1.58) | <0.001 |
| Low SDI | 1.43(1.24-1.63) | <0.001 | -1.07(-1.15--0.99) | <0.001 | -0.93(-1.05--0.82) | <0.001 |
| **East Asia** | | | | | | |
| China | 1.47(0.98-1.96) | <0.001 | -3.09(-3.59--2.59) | <0.001 | -2.26(-2.62--1.89) | <0.001 |
| **Central Asia** | | | | | | |
| Armenia | 2.20(1.96-2.45) | <0.001 | 0.85(0.61-1.09) | <0.001 | -0.08(-0.27-0.12) | 0.423 |
| Azerbaijan | 0.84(0.35-1.33) | 0.001 | -0.04(-0.45-0.37) | 0.835 | -0.85(-1.25--0.44) | <0.001 |
| Georgia | -0.44(-0.72--0.16) | 0.003 | -2.76(-3.16--2.36) | <0.001 | -2.13(-2.47--1.79) | <0.001 |
| Kazakhstan | 0.54(0.20-0.88) | 0.003 | -0.38(-0.67--0.08) | 0.015 | -0.59(-1.04--0.15) | 0.011 |
| Kyrgyzstan | -1.62(-1.82--1.41) | <0.001 | -2.56(-2.70--2.43) | <0.001 | -3.09(-3.36--2.81) | <0.001 |
| Mongolia | -0.35(-0.49--0.21) | <0.001 | -1.26(-1.38--1.14) | <0.001 | -1.34(-1.47--1.20) | <0.001 |
| Tajikistan | -0.26(-0.75-0.24) | 0.293 | -1.27(-1.54--0.99) | <0.001 | -2.91(-3.24--2.58) | <0.001 |
| Turkmenistan | -0.54(-1.07--0.01) | 0.045 | -1.98(-2.45--1.51) | <0.001 | -3.03(-3.56--2.49) | <0.001 |
| Uzbekistan | -1.03(-1.27--0.78) | <0.001 | -2.76(-2.99--2.53) | <0.001 | -1.83(-2.05--1.60) | <0.001 |
| **South Asia** | | | | | | |
| Bangladesh | 3.20(3.07-3.33) | <0.001 | -1.85(-2.20--1.49) | <0.001 | -3.02(-3.40--2.65) | <0.001 |
| Bhutan | 1.67(1.48-1.87) | <0.001 | -1.14(-1.22--1.06) | <0.001 | -1.93(-2.03--1.83) | <0.001 |
| India | 3.49(3.04-3.94) | <0.001 | -0.49(-0.99-0.01) | 0.055 | -0.78(-1.18--0.37) | 0.001 |
| Nepal | 3.87(3.67-4.08) | <0.001 | -1.25(-1.53--0.97) | <0.001 | -1.67(-2.15--1.18) | <0.001 |
| Pakistan | 2.02(1.83-2.21) | <0.001 | 0.48(0.31-0.66) | <0.001 | -2.25(-2.40--2.10) | <0.001 |
| **Southeast Asia** | | | | | | |
| Cambodia | 3.54(3.28-3.80) | <0.001 | -0.36(-0.49--0.23) | <0.001 | 0.36(0.22-0.49) | <0.001 |
| Indonesia | 3.42(3.22-3.62) | <0.001 | 0.27(0.08-0.45) | 0.008 | -0.13(-0.27-0.00) | 0.057 |
| Lao | 3.70(3.53-3.87) | <0.001 | -0.62(-0.74--0.50) | <0.001 | -0.91(-1.11--0.70) | <0.001 |
| Malaysia | 0.16(-0.13-0.44) | 0.271 | -2.13(-2.41--1.85) | <0.001 | -3.24(-3.54--2.95) | <0.001 |
| Maldives | 1.88(1.74-2.01) | <0.001 | -1.16(-1.60--0.72) | <0.001 | -2.45(-2.86--2.03) | <0.001 |
| Burma | 4.10(3.76-4.43) | <0.001 | 0.41(0.34-0.47) | <0.001 | -2.64(-2.76--2.52) | <0.001 |
| Philippines | 1.10(0.88-1.32) | <0.001 | -1.12(-1.28--0.96) | <0.001 | -1.58(-1.83--1.34) | <0.001 |
| Sri Lanka | 1.46(1.31-1.60) | <0.001 | -0.19(-0.34--0.03) | 0.018 | -2.70(-2.85--2.54) | <0.001 |
| Thailand | 1.54(1.19-1.90) | <0.001 | -0.53(-0.83--0.23) | 0.001 | -2.79(-2.94--2.64) | <0.001 |
| Viet Nam | 5.06(4.83-5.29) | <0.001 | 2.91(2.58-3.25) | <0.001 | 0.81(0.77-0.85) | <0.001 |
| **High-income Asia pacific** | | | | | | |
| Brunei | 1.26(1.09-1.42) | <0.001 | -0.74(-0.81--0.67) | <0.001 | -3.00(-3.24--2.76) | <0.001 |
| Singapore | -0.94(-1.25--0.64) | <0.001 | -1.55(-1.96--1.13) | <0.001 | -5.25(-5.80--4.70) | <0.001 |
| **North Africa and Middle East** | | | | | | |
| Afghanistan | 1.09(0.44-1.76) | 0.002 | -0.74(-0.84--0.64) | <0.001 | 1.17(0.95-1.39) | <0.001 |
| Bahrain | -0.69(-0.79--0.58) | <0.001 | -0.86(-1.01--0.71) | <0.001 | -2.54(-2.71--2.36) | <0.001 |
| Egypt | 0.94(0.86-1.03) | <0.001 | 1.08(0.83-1.33) | <0.001 | 0.11(0.05-0.17) | 0.001 |
| Iran | 0.12(-0.10-0.34) | 0.261 | -1.52(-1.81--1.23) | <0.001 | -2.63(-2.77--2.49) | <0.001 |
| Iraq | -1.51(-1.62--1.40) | <0.001 | -2.22(-2.35--2.08) | <0.001 | -3.65(-3.76--3.53) | <0.001 |
| Jordan | 0.62(0.56-0.68) | <0.001 | -0.70(-0.83--0.57) | <0.001 | -1.48(-1.65--1.32) | <0.001 |
| Kuwait | 0.54(0.45-0.63) | <0.001 | 0.71(0.48-0.94) | <0.001 | -1.68(-1.82--1.53) | <0.001 |
| Lebanon | 0.87(0.78-0.96) | <0.001 | -0.13(-0.25--0.01) | 0.029 | 0.31(0.20-0.42) | <0.001 |
| Oman | 3.67(3.52-3.82) | <0.001 | -0.05(-0.41-0.32) | 0.795 | -0.50(-0.97--0.04) | 0.035 |
| Palestine | -0.04(-0.31-0.23) | 0.745 | -1.09(-1.19--0.99) | <0.001 | -1.49(-1.58--1.40) | <0.001 |
| Qatar | 1.13(0.99-1.27) | <0.001 | -0.25(-0.47--0.03) | 0.029 | 0.49(0.26-0.73) | <0.001 |
| Saudi Arabia | 2.22(2.08-2.37) | <0.001 | 0.62(0.47-0.78) | <0.001 | 0.79(0.62-0.95) | <0.001 |
| Syrian Arab Republic | 0.73(0.65-0.82) | <0.001 | -1.27(-1.34--1.19) | <0.001 | -1.78(-1.84--1.72) | <0.001 |
| Turkey | 0.11(-0.19-0.40) | 0.462 | -1.69(-2.16--1.22) | <0.001 | -1.47(-1.71--1.23) | <0.001 |
| United Arab Emirates | 0.42(0.14-0.69) | 0.004 | -1.27(-1.44--1.10) | <0.001 | -0.94(-1.11--0.76) | <0.001 |
| Yemen | 1.35(1.16-1.53) | <0.001 | -1.96(-2.09--1.84) | <0.001 | -1.40(-1.45--1.34) | <0.001 |
| **Central Europe** | | | | | | |
| Albania | 1.14(0.95-1.34) | <0.001 | 0.46(0.28-0.65) | <0.001 | -0.91(-1.16--0.65) | <0.001 |
| Bosnia and Herzegovina | 1.52(1.34-1.69) | <0.001 | 0.31(0.12-0.49) | 0.002 | 0.39(0.31-0.47) | <0.001 |
| Bulgaria | -0.99(-1.19--0.79) | <0.001 | -0.19(-0.32--0.05) | 0.008 | -1.65(-1.75--1.54) | <0.001 |
| Croatia | -0.77(-0.90--0.63) | <0.001 | -0.08(-0.23-0.07) | 0.283 | -2.82(-3.07--2.57) | <0.001 |
| Czechia | 0.21(-0.02-0.45) | 0.071 | -0.59(-1.02--0.16) | 0.008 | -1.01(-1.28--0.74) | <0.001 |
| Hungary | -0.84(-0.94--0.74) | <0.001 | -1.09(-1.30--0.88) | <0.001 | -2.08(-2.19--1.97) | <0.001 |
| Montenegro | 1.10(0.79-1.40) | <0.001 | 0.99(0.71-1.27) | <0.001 | 0.93(0.69-1.17) | <0.001 |
| Macedonia | -1.16(-1.47--0.84) | <0.001 | -0.56(-0.89--0.23) | 0.002 | -2.41(-2.75--2.08) | <0.001 |
| Poland | -2.60(-2.89--2.31) | <0.001 | -3.44(-3.85--3.02) | <0.001 | -4.75(-5.09--4.40) | <0.001 |
| Romania | 0.30(0.25-0.35) | <0.001 | -2.18(-2.42--1.93) | <0.001 | -1.49(-1.58--1.40) | <0.001 |
| Serbia | 0.06(-0.14-0.26) | 0.565 | -0.24(-0.49-0.01) | 0.059 | -0.67(-0.79--0.55) | <0.001 |
| Slovakia | 0.33(0.22-0.44) | <0.001 | -0.20(-0.52-0.12) | 0.211 | -1.21(-1.35--1.06) | <0.001 |
| Slovenia | -0.66(-0.73--0.59) | <0.001 | -0.83(-1.04--0.63) | <0.001 | -2.2(-2.51--1.90) | <0.001 |
| **Eastern Europe** | | | | | | |
| Belarus | -1.77(-2.01--1.53) | <0.001 | -2.15(-2.42--1.88) | <0.001 | -3.05(-3.26--2.84) | <0.001 |
| Es-nia | -0.90(-1.28--0.53) | <0.001 | -2.62(-3.34--1.89) | <0.001 | -2.69(-3.24--2.13) | <0.001 |
| Latvia | -1.6(-1.88--1.31) | <0.001 | -2.39(-2.69--2.10) | <0.001 | -3.45(-3.82--3.08) | <0.001 |
| Lithuania | -0.86(-1.07--0.65) | <0.001 | -1.38(-1.67--1.09) | <0.001 | -2.22(-2.58--1.86) | <0.001 |
| Moldova | -0.65(-0.96--0.34) | <0.001 | -2.72(-3.02--2.43) | <0.001 | -2.06(-2.27--1.84) | <0.001 |
| Russian Federation | -2.75(-2.92--2.58) | <0.001 | -3.00(-3.10--2.90) | <0.001 | -3.37(-3.58--3.15) | <0.001 |
| Ukraine | -2.51(-2.89--2.13) | <0.001 | -2.70(-3.09--2.31) | <0.001 | -3.18(-3.59--2.78) | <0.001 |
| **Western Europe** | | | | | | |
| Cyprus | 0.80(0.62-0.98) | <0.001 | 0.04(-0.14-0.24) | 0.632 | -0.63(-0.73--0.53) | <0.001 |
| Greece | -0.01(-0.11-0.10) | 0.901 | -1.11(-1.24--0.97) | <0.001 | -0.96(-1.05--0.88) | <0.001 |
| Israel | -0.88(-1.05--0.71) | <0.001 | -0.47(-0.81--0.12) | 0.010 | -3.14(-3.31--2.96) | <0.001 |
