# Supplementary figures and images for: Trends in disease burden and risk factors of asthma from 1990 to 2019 in Belt and Road Initiative countries: evidence from the Global Burden of Disease Study 2019
Source: Ann Med. 2024 Sep 6;56(1):2399964. doi: 10.1080/07853890.2024.2399964 (PMC11382694; doi:10.1080/07853890.2024.2399964)

**Figure S1.** Trends regarding the age-standardized rates of YLDs


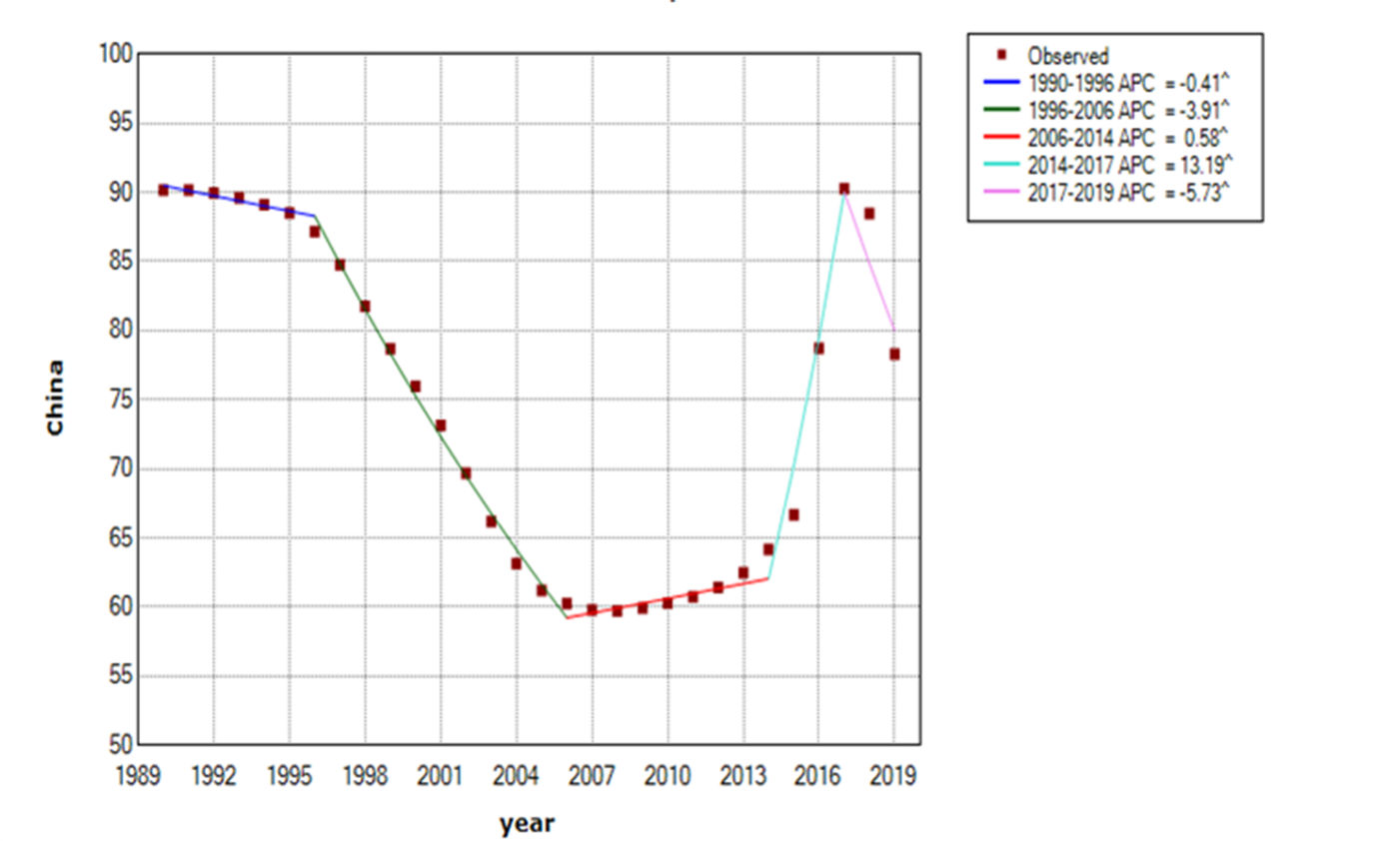

Supplement: Supplemental Material [file IANN_A_2399964_SM5095.zip › Supp_data/SFigure.docx]
